# Touch, communication and affect: a systematic review on the use of touch in healthcare professions

**DOI:** 10.1186/s13643-025-02769-4

**Published:** 2025-02-14

**Authors:** Raffaele Andrea Buono, Minna Nygren, Nadia Bianchi-Berthouze

**Affiliations:** UCL Interaction Centre (UCLIC), University College London, 66-72 Gower St, London, WC1E 6EA UK

**Keywords:** Touch, Healthcare, Instrumental touch, Expressive/affective/socialtouch, Communication, Affect, Adaptive touch, Nursing, Allied health professions, Medicine

## Abstract

**Background:**

The following systematic review explores the uses and understandings of physical, human-to-human touch engagements in healthcare professions. Given its central importance as both a diagnostic tool and a form of non-verbal communication, this review sought to understand the communicative, social and affective dimensions of touches a part of healthcare, medical or nursing interventions. We attempt to understand how touch communication seems to be structured in the literature, and what tends to be communicated via touch, but also to highlight how the dogmatic distinction between ‘instrumental’ and ‘expressive’ touches might have obscured a socio-affective matrix within all touches.

**Methods:**

The synthesis produced was informed by 36 empirical studies involving either direct observation of touch practices, or recollection and discussion with healthcare professionals. The studies were selected from five databases in March 2022. In order to minimise risks of bias, the corpus was screened by two independent reviewers and underwent quality appraisal through the Mixed Methods Appraisal Tool. The final dataset was then analysed, synthesised and presented according to the principles of thematic synthesis.

**Results:**

We outline how medical touch has been mostly categorised as either ‘instrumental’ or ‘expressive’, with only the latter usually described as serving a communicative purpose, despite its lower incidence. We further highlight how touch seems to be operating across a fragile boundary between ‘reassuring presence’ and ‘control’, and thus requires carefulness by practitioners, and an understanding of boundaries. Then, we describe how the literature presented gender, cultural background and personal preference as elements influencing the use and perception of touch. Lastly, touch-mediated communication has been presented in some of the literature as a co-produced practice based on bodily, affective and contextual mutual attunement. Such an understanding radically reconfigures the patient as an active co-participant, as well as pushing against the conceptual boundary between instrumental and expressive touch, recognising how to affect cuts across human-made dichotomies.

**Conclusion:**

We argue that communication might happen in all instances of touch, while also advocating for empirical work to outline and describe the adaptive physical dynamics (e.g. changes in speed, pressure, temperature) that regulate and alter even medical procedures for communicative purposes. We also discuss the need for social scientists to radically re-conceptualise not only the theoretical scaffolding behind medical touch, but also the methodologies deployed to investigate it—advocating for a renewed attention to bodily and interactional dynamics, particularly through the deployment of (micro-)phenomenological tools, broader ethnographical engagements, or sensors for automatic recognition of bio-signals.

**Limitations:**

The review could be at risk of bias given it sampled only studies written in English, French, Italian, Spanish and Finnish, thus not highlighting potentially different cultural and theoretical perspectives emerging from non-EuroAmerican contexts. Moreover, only 36% of studies included discuss patients’ perspectives.

**Systematic review registration:**

This review was not registered.

## Introduction

Tactile perception plays a central role in our ability to engage with the world [1, 2], as it functions not only as a manipulation tool but also as a form of non-verbal communication (NVC) [3]. In light of this, touch provided as part of medical, caring or rehabilitative interventions is of particular analytical interest because, beyond its central role as a tool to examine, diagnose and treat patients, it can also convey a ‘therapeutic sentiment’ ([4]: 4). Touch within healthcare is therefore a complex, multi-faceted communicative channel and process, providing physical, but also psychological and emotional benefits [5]. This stance strongly pushes back against recent calls for ‘hands off’ policies [6]—recognising instead its crucial socio-affective significance [7].

This systematic review thus seeks to highlight this communicative value of touch within healthcare professions, identifying the role and value that touch plays in patient-practitioner communication, as well as identifying further open questions requiring further investigation. This is particularly important considering recent developments in technologies (e.g. social and therapeutical robots) which could support and collaborate with medical professionals in the daily care of patients. Such novel technological interventions might be conceptually radically different from human-to-human touch (see, for instance: [8]), both in terms of how they are perceived, as well as in terms of how they operate. However, a more robust understanding of how medical touch operates beyond merely procedural and diagnostic functions might in turn inform the design of said technologies towards embedding in them similar capacities (e.g. [9–12])—seeing them not as merely supplementary and accessory, but fundamental to successful haptic engagement between patient and practitioner. Still, the present review is solely focused on physical human touch, as it will be further elaborated in the Methods section.

### Background

Studies around medical touch intensified in the late 1950s [13, 14], and much of the work done in the following two decades fundamentally shaped the theoretical lens through which the topic is analysed to this day. Particularly, a dichotomous distinction began to emerge through the work of Wilbur Watson [15, 16]: as a matter of fact, in his sociological study of geriatric nursing, he identified wide variation in the purpose of touches provided by caregivers, ultimately constructing two general forms of touch—i.e. instrumental touch, and expressive touch. On the one hand, instrumental touch refers to physical contact used for the purpose of performing a specific caring task, such as bathing and feeding [17], or administering medications and drawing blood [18]. On the other hand, expressive touch is understood as spontaneous and emotional acts concerned with communicating affective meaning to patients—such as hugging, stroking, patting on the back, or resting touches [19].

Studies on touch in healthcare have mostly reinforced and supported this division, by either: (a.) adhering to it (e.g. [20–22]); (b.) slightly adjusting it with cognate terminologies; (c.) supplementing it with further touch types.

As far as (b.) is concerned, [23] used the concepts of procedural and non-necessary touch, while [24] referred to procedural and non-procedural touch, and [25] spoke of work/task touch, and caring/social touch. Regarding (c.), [26] divided medical touch into spontaneous, pragmatic and silent touches, while [27] described four types of nursing touches: affectional, functional, protective and non-physical. Furthermore, [28] identified caring touch, task touch and protective touch, and [29] divided touch into spontaneous, procedural, non-procedural and investigative. Despite their variety, all these conceptualisations rest upon the assumption that the medical and the communicative happen in separate instances, and, as it will be highlighted throughout this review, these dichotomous taxonomies have been rarely problematised (e.g. [30]).

Compared to the relatively few studies on the use of touch in healthcare professions, many reviews of the topic have been redacted. However, the dataset in this review presents between 25 and 36 new studies compared to the previous reviews summarised below. Furthermore, most of the reviews below focused on one specific care profession (e.g. nursing, occupational therapy), rather than exploring contextual intricacies of touch, or inter-discipline differences.

In their review, [31] analysed types of affective and social touch they defined as ‘interpersonal touch’, excluding instrumental and medical procedures. Moreover, the review solely focused on how interpersonal touch is modulated to reduce stress in ICU patients. Similarly, [32] explored the clinical, measurable effectiveness of touch intervention, rather than exploring subjective benefits highlighted by patients and practitioners. Routasalo [33] instead focused on cataloguing how touch has been conceptualised, and the extent to which different types of touches are used, with little interest in the benefits and communicative aims of said touches. Similarly, [34] collated studies understanding touch as a medical tool, in order to better understand how touch is conceptualised and learnt, with the aim of advocating for more and better training. Ingham [35] reviewed contextual factors shaping and affecting the use of touch, with little concern over the actual touches being performed and the aims of said touches. Similarly, [36] focused on factors facilitating touch. Furthermore, two reviews in the occupational therapy literature [37, 38] focused solely on instrumental manipulations to provide scoping guidelines for practitioners (i.e., meta-aggregation approach), rather than stimulating new research streams on the topic.

Lastly, we would like to single out [39], since we believe it most closely aligns with our analytical interests. Their review is an interpretivist reading [40] of the literature on touch, in order to argue that the body is not merely an object of scrutiny via touch, but it is rather an interactional materiality through which humans engage with each other and the world. From this review, we reprise some of the themes, but instead of rejecting the dichotomy between instrumental and expressive touch from the onset, we focus on examining these conceptualisations as they emerge in the literature. We do this in order to offer a coherent view of touch that, through acknowledging what this division obscures, also highlights why this division might matter, and what we can learn from differing approaches—one focused on cataloguing and dividing, and one understanding touches holistically and beyond boundaries. This seems plausible given the recent call to develop an interdisciplinary understanding of touch, and not least in the light of novel technology development within healthcare practice and training, which poses entirely new questions regarding how to conceptualise and even replicate touch within and beyond human-to-human touch (e.g. [41]), supported by recent empirical research on how technology may contribute to the shaping of ‘nuanced emotional dialogues’ ([42]: 34).

### Aim

In light of this brief background analysis, the present review sets itself apart from previous ones by attempting to understand the communicative, social and affective dimensions of touch in healthcare and nursing professions, in both its instrumental and expressive instances. Our approach provides a comprehensive overview of the phenomenon without privileging one type of touch over another and teases out conceptual and methodological issues that might have been partly obscured in the studies examined and overlooked in previous reviews. In so doing, we foreground how such critical (dis-)junctures point us to a reformulation of touch as *caring* and *care-ful*, incorporating and pushing beyond traditional divisions between instrumental and expressive, and instead embracing rather novel re-conceptualisations of care and affect (see, for instance [43, 44]). This is something we will more organically touch upon in the Discussion, where we approach the questions below from a much broader perspective which includes more theoretical work, or work produced outside of healthcare contexts, in order to contextualise and critique our findings, as well as considering novel directions and methodologies to investigate touch in healthcare.

In order to achieve this aim, this paper analyses studies in healthcare settings exploring how practitioners (and patients) use, experience, and understand touch when providing care. The analysis is guided by the following questions: (1) what is communicated via touch?; (2.) what touch instances tend to be seen as communicative?; (3.) what influences the use, as well as the communicative effectiveness, of touch?; (4.) how is touch-mediated communication structured?; (5.) how have communicative elements of medical touch been picked up in the literature?

## Methods

### Dataset identification

The first author of this review ran a comprehensive search across five databases in March 2022: SSCI, MEDLINE, ACM, CINAHL and OTSeeker. Some of these databases (i.e. SSCI, MEDLINE, CINAHL, OTSeeker) were selected for their coverage of the subject matter (i.e. social scientific analyses of healthcare practices); ACM was added in an attempt to find studies which compared human touch to non-human touch in healthcare. The search was *planned—*i.e. all databases were consulted, and the final review corpus was compiled before the actual analysis commenced.

A PICo (Population, Interest, Context) framework ([45]; see also: [46]) was used to formulate the preliminary question ‘What are care-givers’ (i.e. allied health professionals, nurses, doctors, carers) [Population] actual uses and experiences of touch [Interest] when providing real and direct medical care to patients [Context]?’. From this question, PICo was also used to devise inclusion and exclusion criteria and to inform an initial set of search terms. The search results and selection process outlined below follow the PRISMA 2020 standard [47] and the ENTREQ checklist [48] (Appendices 1, 2, and 3).

**Fig. 1:**
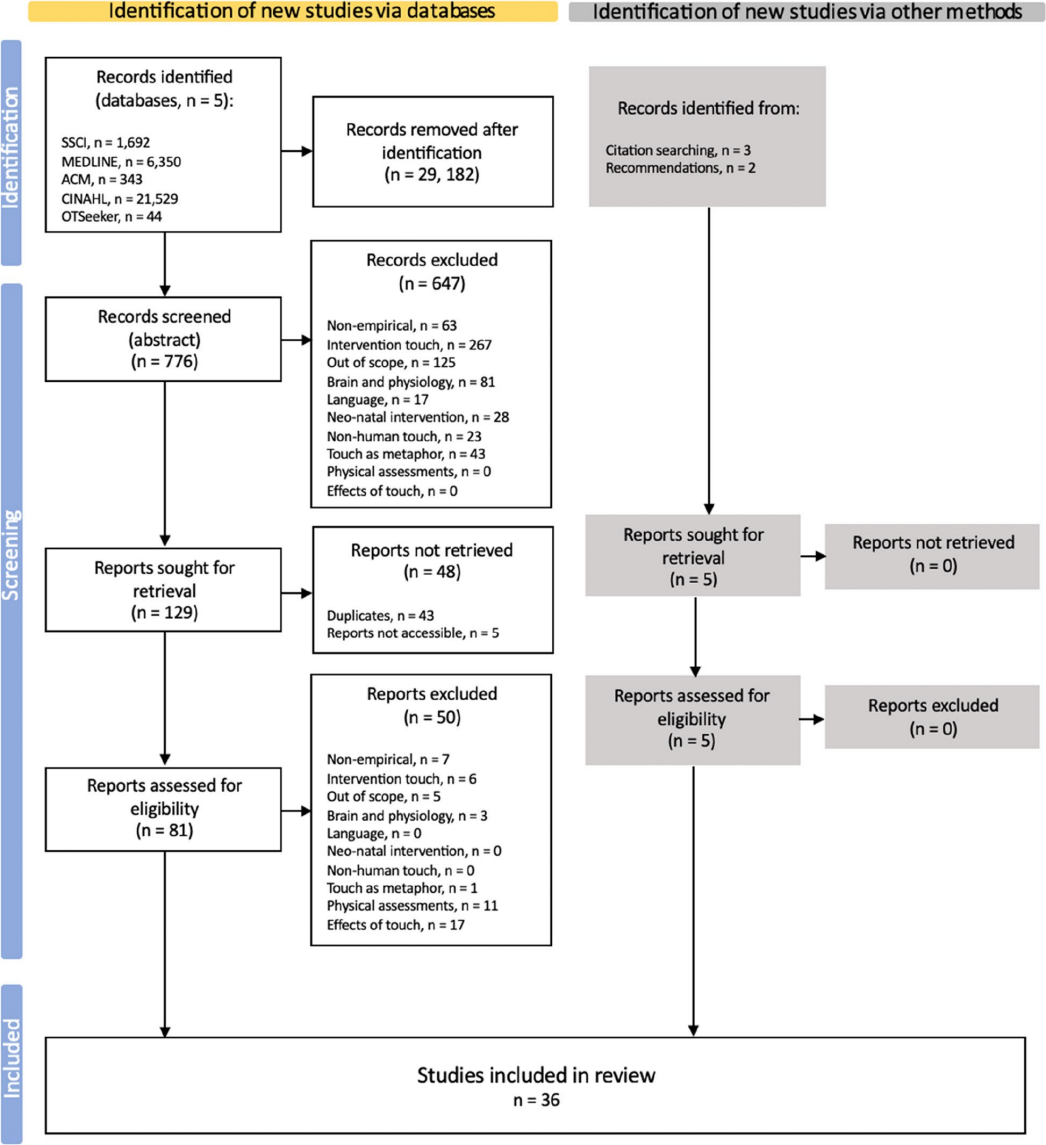
Corpus identification flowchart

These initial queries produced 29,958 results. Selection has been performed by one human (first author of this manuscript) during the first and second stages (screen-by-title, and screen-by-abstract), and by two researchers in the third (screen-by-text), without the support of automated tools. As detailed in Fig. 1, these results were reduced to 776 studies after title screening: studies at this stage were excluded merely by gauging whether they actually dealt with touch. In this sense, because our search strategy included incredibly common terms such as ‘experience’ and ‘touch’, which can be used in very different contexts from what we envisaged (e.g. ‘to touch upon’ meaning ‘to mention’), a large number of papers (*n* = 29,182) was excluded at this preliminary identification stage.

The remaining studies were then screened by abstract, applying the specific exclusion criteria outlined via PICo: 647 studies were excluded, leaving 129 records to retrieve for full-text screening. Of these, 5 could not be accessed, and 43 were duplicates. 81 records were thus retrieved.

After an initial reading of the 81 studies remaining, to both better outline the research aims of the present review, and to get an initial appraisal of the quality of said papers and their relevance to the aims of this review, a full-text screening was performed. In order to minimise risks of bias, a conventional double-screening approach [49] was followed at this stage, with the original reviewer and a second reviewer independently assessing the dataset. After discussing disagreements, the corpus contained 31 studies.

Lastly, three studies were identified and added to the corpus by screening references in previous reviews. Two studies recommended by two field experts who had been contacted for guidance over the dataset selection have also been added.

The finalised corpus comprised 36 studies. The results of the appraisal for each study included in this synthesis can be found in Appendix 6.

### Inclusion/exclusion criteria and quality appraisal

The present review is exploratory in nature: for this reason, studies from all years and in any language spoken by the team (i.e. English, Italian, French, Spanish, Finnish) were included (‘Language’ exclusion criterion). Both qualitative and quantitative studies were considered eligible, but non-empirical studies were not (‘non-empirical’ exclusion criterion). No specific sociometric variables (e.g. sex, age, socioeconomic status of participants) were sought, and studies were not excluded on the basis of their design (e.g. number of participants, methods).

While the review aimed to capture as broad a dataset as possible, 10 exclusion criteria have been used to parse through the corpus (Table 1). Two of them (‘language’ and ‘non-empirical’) were based on the team expertise and the general aim of this review (i.e. capturing empirical social analysis over anecdotal case studies or philosophical discussions), while the other 8 were formulated starting from the PICo framework.

**Table 1:** Exclusion criteria

Exclusion criteria		Excluded (abstract)	Excluded (full-text)	Reason
**Non-empirical**		*n* = 63	*n* = 7	Articles not presenting empirical evidence (e.g. opinion pieces, commentaries, guidelines), as well as individual case studies providing anecdotal evidence
**Language**		*n* = 17	*n* = 0	Articles not in English, Italian, Spanish, French, Finnish
**Population** **(P)**	Non-human touch	*n* = 23	*n* = 0	Articles presenting touch performed by autonomous technological agents (e.g. robots), or in virtual environments, rather than via direct skin-to-skin contact
	Out of scope	*n* = 125	*n* = 5	Articles presenting findings from outside the standard remit of allied health professions, nursing, medicine, and care work
**Interest** **(I)**	Brain and physiology	*n* = 81	*n* = 3	Studies investigating how touch functions in terms of brain processing dynamics, or touch physiology
	Touch as metaphor	*n* = 43	*n* = 1	Articles using touch terminology as a metaphor to describe emotional engagement, e.g. ‘being touched by the kindness of a nurse’, or to discuss embodiment in general terms, with touch being just one of many embodied strategies
	Effects of touch	*n* = 0	*n* = 17	Articles describing in general terms how touch can benefit (or hinder) patients, without actually describing what specific touch instances do what
	Physical assessments	*n* = 0	*n* = 11	Articles focusing solely on the practice of touch in terms of biomechanics, rather than on its socio-cultural elements
**Context** **(Co)**	Neo-natal interventions	*n* = 28	*n* = 0	Articles presenting data referring to touch interventions towards pre-verbal children, for whom touch has different properties, and because of the different anatomical conformation of babies
	Intervention touch	*n* = 267	*n* = 6	Articles presenting forms of touch and touch techniques for therapeutic, non-medical relief (e.g. Reiki, energy practice, message therapy), since these forms of touch differ conceptually from physical touch for the purpose of clinical support (instrumental or affective) [50]

In particular:

1. *Population*: exclusion criteria ‘Non-human touch’ and ‘Out of scope’ were devised to include only studies exploring human-to-human touch in allied health professions, nursing, medicine and care work;
2. *Interest*: exclusion criteria ‘Brain and physiology’, ‘Touch as metaphor’, ‘Effects of touch’ and ‘Physical assessments’ were used to include only studies describing actual instances of touch as part of a medical intervention, describing not simply the biomechanical, physiological or neurological component of said touch, but also some of the social, cultural and professional context within which it had happened;
3. *Context*: exclusion criteria ‘Neo-natal intervention’ and ‘Intervention touch’ were applied to include only studies dealing with instances of routine medical touch on patients able to verbalise their reactions.

At the full-text stage, papers underwent a quality appraisal and risk of bias analysis through the Mixed Methods Appraisal Tool (MMAT) [51, 52] (Appendix 6); MMAT was preferred over tools such as the Standard for Reporting Qualitative Research (SRQR) [53], given our synthesis included qualitative, quantitative and mixed methods studies. Papers were appraised by two reviewers for *methodological robustness*, and for their *utility* for the present review. Consensus was required for a study to be considered eligible for review, so the two reviewers discussed disagreements to resolve them. No papers were excluded because of methodological quality, but 50 additional studies were excluded on the grounds of the exclusion criteria used.

For instance, studies such as those of [54, 55] and [56] initially appeared to respond to the question posed by the present review; upon a closer appraisal however, they were all excluded because they focused solely on assessing the functional dynamics and efficacy of touch interventions, without exploring the impact of such a touch experience on either the practitioner or the patient. Moreover, studies such as [57] were excluded because they solely focused on what touch does, without enquiring into what these haptic engagements are, as well as when how they happen in the specific medical context analysed: in this sense, while the medical context is evinced from these studies, it does not end up mattering in their analyses.

Lastly, a number of studies rooted in *conversation analysis* (CA) appeared in the initial search but were excluded for different reasons. For example, [58] and [59] develop interesting conceptual descriptors to understand the role of touch in social interactions, but do so in contexts outside of the remit of this review (i.e. interpersonal relations among friends, and parent–child dyads, respectively). On the other hand, work such as [60–62] remain within the context of healthcare, but analyse touch only in passing, as one of many resources across the ‘body language’ conceptual spectrum, without enquiring regarding the specificities of touch. Some of this work in this sense foregrounds some novel conceptualisations that will be more robustly operationalised in the “Discussion” section. However, they had to be excluded from the actual corpus, given the focus of this systematic review, and hence its specific search criteria.

### Data extraction and methodology

Table 2 provides a summary of the characteristics of the included studies. Once the final review corpus of 36 studies was compiled, the first author extracted data without the use of software, aside from a standard word processor and spreadsheet editor. The data extraction, analysis and generation took a bottom-up approach: while the general direction of the review was established at the initial stage, the data analysis stage was not bound by any deductive framework. Instead, themes were constructed inductively, with new codes and themes created as necessary. Furthermore, the review followed the principles of *thematic synthesis* [63], identifying patterns and themes with the aim of uncovering underlying and often covert issues (i.e. communication and affect via touch) in order to generate new critical insights and directions (see “Discussion” section). Thematic synthesis was preferred over meta-aggregation for two main reasons. First, most of the reviews on the topic generated thus far have focused on providing descriptive accounts to guide practitioners and policymakers. Second, and conversely, our interest lays in uncovering, exploring and understanding underlying theoretical assumptions regarding the subject matter: such an aim thus required a methodological toolkit that allowed for flexibility in critical interpretation to generate novel critical insights and directions from the identification, evaluation and discussion of implicit narratives and approaches in the literature. In this sense, and as will be evident in the discussion, the aim of this review was that of opening new pathways for the study of touch, rather than providing guidelines for practitioners.

**Table 2:** Study characteristics

Authors	Year	Country	Participants	Context	Methods	Research questions
Adomat and Killingworth	1994	England	60 nurses	Nursing (ITU)	Mixed methods Non-participant observation Semi-structured interviews	Do nurses with more than 2 years FT experience in ITU communicate less caring touch to patients?
Barnett	1972	USA	540 patients 900 practitioners	Healthcare	Quantitative Non-participant observation	What types of touch are used in different hospital wards? What body parts are touched more often? How do age, race, sex of the toucher and touched, as well as economic and social status of the patient affect touch?
Bjorkbækmo and Mengshoel	2016	Norway	9 PT/user dyads	Physiotherapy	Qualitative Phenomenological Non-participant observation Semi-structured interviews	What is the ‘body’ being touched in PT? What is being touched like in PT? What does being touched imply in PT?
Bundgaard et al	2011	Denmark	8 patients 4 nurses	Nursing (short-term stay facilities)	Qualitative Phenomenological Participant observation Unstructured interviews Participants’ reports	How is caring touch understood by practitioners and patients? Are there differences in caring touch usage in short-term facilities, compared to long-term?
Bunzel et al	2020	Denmark	10 nurses	Nursing (ICU)	Qualitative Semi-structured interviews	How are minimally sedated patients moved? What kind of communication happens via touch?
Caris-Verhallen et al	1999	Netherlands	47 nurses	Nursing (elderly care)	Quantitative Observation of video-recorded nurse-patient encounters	To what extent do nurses use non-verbal communication? How is NVC related to VC? Is NVC related to the setting (home for the elderly) and the kind of care provided?
Cocksedge et al	2013	England	15 GPs 11 patients	General medicine	Qualitative Semi-structured interviews (doctors and patients)	How is touch used in primary care consultations? How does it change once an ongoing relationship is established?
Consedine et al	2016	New Zealand	5 osteopaths	Osteopathy	Qualitative Phenomenological Semi-structured interviews	How can the experience of being touched and touching during an osteopathic session be qualified?
De Carvalho de Rezende et al	2015	Brazil	9 nurses	Nursing	Quantitative Questionnaires Observation	What are the kinds of body language and touch gestures used by nurses to communicate with patients?
De Luca et al	2021	Italy	39 nurses	Nursing	Qualitative Workshops	How do nurses feel about interpersonal touch during nursing care? Do nurses explicitly trained on touching have different attitudes towards it than those who did not?
De Luca et al	2022	Italy	22 nurses	Nursing	Qualitative Focus groups Semi-structured interviews	How do nurses use touch as part of their practice? What kinds of valence does it have, beyond the procedural, and how can touch be effectively incorporated into holistic interventions?
Dobson et al	2004	England	31 healthcare practitioners	Care work	Qualitative Focus groups	What are carers’ awareness, attitudes, and values regarding their touch towards service users?
Eber	2018	Germany	12 care workers 5 children (11–17)	Care work (residential care)	Qualitative Participant observation	How is the agency of children and young adults in residential care produced during and through everyday practices of care involving touch?
Edwards	1998	England	7 nurses	Nursing (ICU)	Qualitative Participant observation	How do nurses use and interpret their use of touch?
Estabrooks and Morse	1992	Canada	8 nurses	Nursing (ICU)	Qualitative Interviews Workshop	How do nurses learn how to touch, and develop a touching style? How do nurses perceive touch and the process of touching?
Gale and Hegarty	2000	England	9 clients	Care work (learning disabilities)	Quantitative Non-participant observation	How do carers for people with learning disabilities use touch? What types of touch are used? Where are clients usually touched? How do clients interpret such touch? How frequently do care staff touch clients during everyday caring? Do trained nursing staff touch differently? How much touch is functional/instrumental, and how much is expressive/affective?
Gleeson and Higgins	2009	Ireland	10 nurses	Nursing (psychiatry)	Qualitative Semi-structured interviews	What are psychiatric nurses’ views and perceptions on their use of touch on people who experience mental health breakdowns?
Hollinger and Buschmann	1993	USA	100 nursing home residents 100 caregivers	Care work (elderly)	Quantitative Questionnaires	How do elderly nursing home residents and health caregivers perceive touch? What attributes influence their perception of touch? Are there differences in perception between caregivers and residents?
Jung and Fouts	2011	Central Africa	35 children	Care work	Quantitative Non-participant observation	Among Bofi foragers, what differences are there between the touch interactions between children and different caregivers?
Karlsson et al	2022	Sweden	13 nurses	Nursing (ICU)	Qualitative Semi-structured interviews	What is the meaning of the caring touch provided by ICU nurses to patients, from the perspective of the healthcare professionals themselves?
Kelly et al	2019	Canada	15 physicians	General medicine	Qualitative Phenomenological Unstructured interviews	What are physicians’ experiences of communicating via touch? How can touch be taught in a medical education curriculum?
Kelly et al	2020	Canada	6 clinicians	General medicine	Qualitative Co-operative inquiry	How do physicians experience communicative touch in clinical practice?
Leonard and Kalman	2015	USA	11 patients	Nursing (oncology)	Qualitative Phenomenological Semi-structured interviews	How do patients experience being touched when receiving chemotherapeutic treatment for cancer?
McCann and McKenna	1993	Northern Ireland	14 patients	Nursing (elderly care)	Mixed methods Semi-structured interviews Non-participant observation	What is the amount and type of touch received by elderly patients from nurses? How do patients perceive the touch that they receive?
Mononen	2019	Finland	Not specified (involving both caregivers and patients)	Care work (elderly care)	Qualitative Non-participant observation Micro-interactional analysis	How do caregivers use affective touch as a resource to facilitate and stimulate socio-affective interaction?
Morris et al	2014	USA	33 OTs	Occupational therapy	Quantitative Non-participant observation OT Interaction Assessment instrument	What are the types of touch, and their frequency of utilization, deployed by OTs when providing care to users?
Mulaik et al	1991	USA	98 patients	Nursing	Quantitative Questionnaires Patient Touch Questionnaire Interpersonal Behaviour Survey	What are patients’ perceptions regarding the amount and kind of touch provided by nurses? What are patients’ beliefs and attitudes towards touch? What are their preferences and responses to touch, and how are these affected by demographic factors?
O’Lynn and Krautscheid	2011	USA	24 laypeople (no nursing training)	Nursing	Qualitative Focus groups	How do patients prefer to be touched, particularly with regard to intimate procedures?
Pedrazza et al	2018	Italy	198 nurses	Nursing	Quantitative Questionnaire	Is there an association between a nurse’s attachment style and their inclination to worry, and their feelings of comfort with touch practices?
Pratt and Mason	1984	England	76 laypeople	Healthcare	Quantitative Questionnaire	What are the intentions of a specific touch gesture in a given scenario?
Roger et al	2002	USA	15 PTs	Physiotherapy	Qualitative Observation Structured interviews using video recordings from PT’s own practice	How do physiotherapists use touch during clinical practices? What aims and meanings do different touches have? What is communicated via touch?
Routasalo	1996	Finland	94 patients 32 nurses	Nursing (elderly care)	Quantitative Non-participant observation	How often, and in what situations, do nurses use non-necessary touch on aged patients? Do the amount vary between morning and evening? Where is touch more used? Do nurses say something while touching a patient?
Routasalo and Isola	1996	Finland	30 nurses 25 patients	Nursing (elderly care)	Qualitative Semi-structured interviews	What do nurses experience when touching elderly patients? How do elderly patients experience being touched?
Routasalo and Isola	1998	Finland	5 nurse/patient dyads	Nursing (elderly care)	Qualitative Phenomenological Non-participant observation	How do skilled nurses in long-term care touch elderly patients who have lost verbal communication ability?
Salzmann-Erikson and Eriksson	2005	Sweden	4 patients	Nursing (psychiatry)	Qualitative Phenomenological Semi-structured interviews	What are the meanings of touch for patients who have been hospitalised for psychosis?
Tarantino et al	2018	Italy	21 patients 14 nurses	Nursing (medicine and surgery)	Qualitative Phenomenological Semi-structured interviews	What are the uses and characteristics of touch in nursing? What are the perceptions, feelings and experiences of both nurses and patients in a touch encounter?

Coding and data extraction was carried out by one reviewer (first author). Salient information was first annotated via a first read-through, with line-by-line coding of each study. These initial codes were discussed with the research team (3 members), with the aim of identifying a set of descriptive themes to guide the following read-throughs. Via a second read, each study was allocated to multiple descriptive themes that emerged while reading the corpus, and consolidated via group discussion. In conformity with guidelines on thematic synthesis [64], through a third read, descriptive themes were connected into 4 higher-level analytical themes. A final read-through and categorisation carried out by the whole team then aimed to explore some of the issues presented in the discussion, as well as to perform a confidence assessment of the themes via the CERQual tool [65], and a heterogeneity analysis of the reported results (Appendix 5). The causes of heterogeneity have been mostly explained via sub-group analysis or methodological evaluation.

## Results

### Themes and literature contextualisation

The findings have been clustered into 4 analytical themes, further sub-divided into specific descriptive sub-themes (see Appendix 4): (I) quantitatively mapping touch; (II) qualitative intents and meanings of touch; (III) touch and its actors; (IV) touch as a waltz.

While these themes partly responded to the general aims of the review outlined above, they were not decided a priori nor used as a frame to analyse the literature. Rather, they were identified from the literature after an iterative and comparative analysis of the corpus, and the initial descriptive themes outlined through an initial analysis.

Before analysing the themes, it is important to contextualise the dataset, given its wide variety in terms of domains of interest, geographical areas and research methods deployed.

As far as fields of enquiry are concerned, an overwhelming majority of the literature concerned itself with touch happening in nursing—i.e. 21 studies out of 36 (58%). The remaining 42% is divided as follows: five studies in care work; two studies in physiotherapy; three studies in medicine; one study in occupational therapy; one study in osteopathy; one study in general healthcare practice.

Even more striking is the lack of geographical variety, with only two studies conducted in a non-Western context—i.e. one study in Central Africa, one study in Brazil [66, 67]. This might be partly influenced by the fact that our dataset search did not include papers written in languages such as Portuguese, Chinese, or Japanese.

It is also worth noting that less than half of the studies under examination (*n* = 15; 42%) attempted to explore patients’ feelings and attitudes regarding touch. Moreover, 2 of these [4, 68] set out to explore patients’ perspectives but did not include any form of direct engagement with patients in their data collection protocol. Thus, it can be stated that only 13 studies (36%) attempted to capture patients’ viewpoints using their own words and conceptualisations.

Lastly, the dataset is comprised of studies following both quantitative and qualitative methodologies. In particular, 23 papers were qualitative, 11 were quantitative, and 2 deployed mixed methods approaches. Within the qualitative studies, 61% (*n* = 14) relied on semi-structured or unstructured interviews, focus groups, or a combination of the two. One additional study took a cooperative inquiry approach. Thus, only 8 studies (34%) involved a researcher actively observing touch instances in a naturalistic setting: out of these, 4 solely deployed ethnographical observation as a data collection method, while 4 combined observation and subsequent interviewing of the participants (i.e. either the medical practitioner performing the touch, or the patient receiving it). Interestingly, among the four studies combining interviewing with direct observation, only [69] developed a protocol allowing the researchers to enquire with participants about the very touches they had observed previously—i.e. video recording touch instances to replay to interviewees. The other three studies [70–72] used the combination of these two methods in a more disjointed way, rarely prompting participants to discuss actual instances observed, but rather questioning them around more generalised practices and motivations.

Furthermore, it is worth noting that most recent studies in the dataset often grounded themselves conceptually within the field of phenomenology (*n* = 8), stressing analytical attention to the body as a site of co-production, and thus bringing forth a renewed attention to embodied, in-the-moment gestures and their affective character.

### Theme I: quantitatively mapping touch

This narrative thread in the literature illustrated the extent to which touch is deployed, as well as in what capacity. The studies thus focused on describing and quantifying how much, how often and in which instances touch is used, which parts of the body tend to be touched, types and expressions of touch being used in allied medical professions, and the extent to which touch is withdrawn under certain circumstances.

#### Touch location on the body

Some studies focused on exploring how different parts of the body are touched in unequal measures, and according to cultural norms of decency and interpersonal privacy. These studies were heavily influenced by work [73] on touch intimacy, which adapted Hall’s [74] theory of proxemics of space. While the latter focused on describing established distances between bodies as constituting different spheres of interpersonal relationship (i.e., the intimate space, the personal space, the social space, the public space), the work of the former instead attempted to understand how touching different parts of the body equates to touching differing zones of intimacy, and thus stimulates different feelings and reactions in both the toucher and the touched. Ebersole and Hess [73] constructed a body taxonomy divided into a social zone (i.e. hands, arms, shoulders, back), a consent zone (i.e. mouth, wrist, feet), a vulnerable zone (i.e., face, neck, front) and an intimate zone (i.e., genitalia).

The studies analysed in this review mostly aligned with the taxonomy above, with most studies highlighting touches in the social zone as happening the most often [20, 75], as well as usually being seen as ‘safe’ [76]. Only [77] highlighted a non-social zone (i.e., the face) as being touched often. Such finding strongly contrasts with the rest of the literature, and particularly qualitative studies (e.g., [76]; [20]) finding that patients feel uncomfortable when being touched in the vulnerable and intimate zones, and would like such touches to be performed only when strictly necessary.

#### Classifying touch across the instrumental and expressive binary

Furthermore, most studies in this cluster attempted to not only quantify touch instances but also to subjectively classify them according to the distinction between instrumental and expressive touch described previously. While they all agree on the fact that instrumental touch seems to be used more often, the extent to which expressive touch is deployed varies. McCann and McKenna [20] for instance, recorded merely seven instances of expressive touch out of 149 touches recorded (4.7%). Conversely, [78] and [79] recognise an expressive touch incidence of 25% and 20% respectively. Lastly, [80] recorded expressive touch in more than 40% of nursing encounters, while also recognising that the amount of time spent performing these kinds of touches is usually only 1–5% of total touch time.

On the one hand, such variability of incidence recorded could be attributed to differences in counting methodologies, or in different nursing contexts in which these studies have been conducted, both culturally, but also medically (i.e., different wards). On the other hand, and more crucially, we could consider these vastly discrepant results as an early indicator of how feeble the definition of expressive touch, as strongly opposed to medical, instrumental ones, is—leading thus to equivocations and differing understandings. In this sense then, it is also unclear if said expressive touches were part of medical procedures and thus served a secondary communicative purpose, or if they were the sole engagements happening.

#### Classifying touch as a-contextual actions

As far as cataloguing *touch actions* [21, 81] is concerned, [78] singled out stroking, rubbing, holding and squeezing as the most frequently recorded; [75] recorded 114 instances of long touches performed with the flat of the hand, 28 instances of patting, 16 of stroking, and less than 10 instances each of shaking, tickling and hugging; [68] instead summarily mentions stroking and embracing as the most common touches. All these instances were categorised as expressive, ‘non-necessary touches’ [75], and the discrepancy in reporting could be attributed to the three very different contexts in which they operated (i.e., learning disabilities care, nursing, care for the elderly).

More interestingly, none of the studies mentioned provided *thick descriptions* [82] of instrumental procedures as they unfolded in situ*:* this seems to prefigure a certain disinterest in describing medical procedures, perhaps because of an assumed lack of details useful to social analysis—i.e. they are seen as nothing more than mechanical manipulations with little to no variation.

Concerning the central aim of touch, [79] developed a recording instrument for the assessment of touch instances in occupational therapy, which included ‘touch aim’ as one of its variables. Through this, their study revealed that 43% of instrumental touches were to support and assist in functional mobility and related exercises, 24% were performed to provide and illustrate instructions, and 17% to adjust equipment. The study did not attempt to categorise and quantify also affective aims of the touches observed.

#### Directionality of touch

Interestingly, only [76] investigated the directionality of touch, highlighting how patients in the ICU expect, and sometimes long for, the touch of nursing staff. Conversely, however, medical personnel do not anticipate being touched by patients, and this reversed direction of touch could destabilise the patient-nurse relationship.

### Theme II: qualitative intents and meanings of touch

Within the following thread, we grouped studies that enquired into and attempted to describe the communicative and affective aims of touch instances, illustrating for instance the meaning of specific touches, the emotions communicated via touch, and the social intents of particular gestures. It is important to preliminarily mention how most studies in this section concerned themselves with expressive instances of touch, or complementary, non-necessary touches alongside instrumental ones, almost implying that the communicative-affective value of instrumental touches is negligible, if not non-existent.

#### Touch as care

First, much of the literature ascribed a double function to touch—simultaneously a ‘tool’ and a ‘resource’ [83]. Touch in this sense does not merely provide factual knowledge to act upon, but can also be leveraged to enhance communication and care [84]. For instance, [80, 83, 85] and [86] all recognise the capacity of non-necessary touches to reaffirm verbalised empathy: gestures such as handshakes pats on the back, hugs, and holding hands can open up a pathway to improving communication quality, with patients experiencing being nurtured, supported, reassured, and respected.

These affective values are also reported in other studies in this cluster. For instance, [4] describes ‘tactile care’ as the ability to convey reassuring and nurturing warmth through touch in instances where words might not be enough, as also mentioned in [87] and [88]. Touch in this sense is seen as an instinctive act, adaptable to different scenarios—for instance, touch is observed to be used as a rapid emotional response to patients’ distress [89].

#### Touch as persuasion and encouragement

Interestingly, [76] also reveals how support-via-touch could also be present as a form of persuasion, for instance by holding patients’ hands and slightly accompanying them to stimulate them towards moving where they need to be. Routasalo [75] and Mononen [68] further build on this power dynamic regulated by touch, observing how nurses and carers often use touch in connection to statements they make to create a framework of empathetic encouragement.

#### Multiple intents

While the work presented thus far focused on expressive touch engagements, some touch instances have been reported to have multiple intents combining task-related and communicative elements. For instance, [69] confirmed the double function of touch as a tool and communicative resource, but subtly shifted the focus towards instrumental engagements (see also *combined touches*: [90]). In this sense, they observed how physiotherapists often do not simply assist and guide through touch, but simultaneously demonstrate care and provide security, for instance by placing their free hand on the patient’s body, despite not being needed from a functional-rehabilitative perspective. Similar considerations have also been drawn by [71] in their observations of nursing procedures. For instance, they observed how nurses often hold patients’ hands while getting intravenous access, or place a hand on their shoulder while performing an endoscopy procedure: these touches are thus part of larger tactile engagements including instrumental procedures. However, it is worth noting how, while *part of* an instrumental procedure, these touches are always supplementary: it is thus implied that the affective-communicative messages happen through these expressive and supplementary touches, while the instrumental engagement remains the same, and devoid of meaning. Tool and resource co-exist in the procedure, but not within the same touch.

#### Touch as opportunity and risk

Touch is also described as feebly existing in a tension between two poles: touch as a humanising presence furthering emotional engagement, and touch as a risk potentially displacing boundaries and safety.

On the one hand, several studies found that touch can foster the establishment of a co-shared human space beyond the aseptic medical setting, a space where affective proximity emerges [86] and empathetic bonds are allowed to flourish [91]. Mononen 68] for instance argued that the hospice carers observed often used gentle strokes and caresses to not simply gather attention to a task, but also to construct a participation framework [92, 93] via haptic co-presence [94]. In this sense, [67] argue that touch adds a humanising dimension to care, creating a sense of trust (see also: [95]): through touch, patients can feel a human presence, they can sense that someone is there ready to help and willing to take care of them [71]. Kelly et al. [96] cogently discuss how trust emerges through this breaking of professional boundaries since touch shows an opening up towards patients’ vulnerabilities and compassion towards them. Besides trust, the emergence of this empathic space fosters a sense of safety and protection in patients, with participants interviewed in some studies (e.g., [97]) going as far as mentioning they long to be touched, because of the protection they feel through it.

On the other hand, several studies reported that touch can also be easily misinterpreted. For instance, patients interviewed in [98] mentioned how touch can indeed demonstrate affection, but when used in the wrong way that affection might be seen as control (see also: [71]). Tarantino et al. [95] in this sense conclude that the inherent risks of touch are amplified when a nurse does not establish a relationship based on mutual proximity and consent, since when these are lacking, even comforting touches can feel deeply distressing. These negotiations mostly happen verbally, or by reading patients’ non-verbal reactions to touch and adapting accordingly. All studies thus agree on the need for touch to be dialogical and open to adaptation [96]—something which will be more organically discussed in the fourth narrative.

### Theme III: touch and its actors

Within this theme, we explore how the literature has discussed the roles that identity, personal lives and professional histories play within touch, both in regard to the person performing the touch, as well as the one receiving it.

#### Touch and professional experience

In mixed contexts, such as a hospital ward, [67] observed that nursing staff tends to touch more and more often than doctors and other healthcare practitioners (see also: [95]). Barnett [77] came to similar conclusions, further hypothesising that medical interns and doctors tend to touch less than nurses and carers because they are trying to uphold and achieve cultural expectations regarding their professional roles.

Another professional element found to have an impact on touch dynamics is that of length of service. In this sense, [25] observations suggest that, while the length of service did not impact the amount of instrumental touch provided, nurses with less than 2 years of experience engaged in significantly more expressive touches than more experienced nurses.

Connected to this, research in physiotherapy (e.g., [69]) suggests instead something slightly different. While experienced physiotherapists might indeed touchless, that does not equate to less communication happening via touch: thanks to accumulated experience, expert physiotherapists are able to communicate more via fewer touches, suggesting that one single touch can have multiple intents as discussed in the previous theme. In this sense, this study in physiotherapy already teases out an important element that will be more organically reprised in the discussion—namely that of the inherent issue of relying too strongly on an approach focused on quantifying and categorising types of touch into tied categories. As a matter of fact, said approaches obscure precisely how, particularly with time and experience, practitioners start to embed in their procedural touches communicative and affective elements, rather than operating on a binary—i.e., manipulating the patient *or* communicating via touch.

#### Touch, gender and age

Aside from the work on familial caregivers in central Africa [66], which found no prominent interaction between touch and gender, all other studies concerned with the topic found important correlations between gender (of both the toucher and the touched) and the types of touch being performed. As a matter of fact, research among occupational therapists [79] revealed that male practitioners used instrumental touch 33% more often than their female colleagues, who in turn used expressive touch twice as often as their male counterparts. These findings are in line with the interviews conducted in [20], in which patients stated they would feel uncomfortable if touched expressively by a male nurse, as well as the interviews with nurses themselves conducted in [99], in which nurses stated that it is challenging for men to touch patients, because of the lingering fear of possible allegations of sexual misconduct: this is particularly important when treating women, in which case male nurses mentioned they tend to touch more sporadically and cautiously. Interviews with patients in [100] further strengthen this point, since both female and male patients stated they prefer being touched by a woman whenever possible, while [98] empirically proved the reticence towards touching female patients, recording that men are touched twice more often.

Within this dataset, [75] stands as an outlier, being the only study observing that female patients receive more touch than men, and that expressive touch is used slightly more often when engaging with female patients. Such a study was carried out in Finland, which might allow us to ascribe the difference to differing cultural attitudes to touch and gender. However, while we can speculate on such questions, no study analysed has extensively discussed higher-level questions related to cultural attitudes to touch.

Age seems to be less of a factor of concern, with only [98] and [77] reporting that the younger the nurse, the more often they will touch a patient, while [85] observed that older patients tend to be touched by practitioners more often. While [25] were the only ones to analyse both age and length of service as variables, they found no statistical relation between age and amount/type of touch. Such a discrepancy could be explained by hypothesising that, when both variables are taken under analysis, length of service has more relevance over age—i.e., inexperienced practitioners could be seen as ‘younger’ (and thus touch more) irrespective of their biological age.

#### Touch, personal preferences and style

A considerable number of studies additionally engaged with questions around personal preferences and individual histories affecting the amount, type and frequency of touch.

With regards to the carer, [89] and [96] observe how touch is most often an act of choice: carers will thus touch patients differently, and according to how they have been socially sensitised to touch. In this sense then, they argue that age, ethnicity and background of a medical professional are variables to take into consideration when examining touch practices. In this sense, [90] sketched out three sequential stages to developing a practitioner’s touching style: one’s own socio-ethno-cultural upbringing, learning experiences in nursing school, and encounters with patients while practising. These three stages interact and influence one another, leading to each nurse developing their own preferences, thus making it hard to classify touch styles into neat categorisations. Moreover, [87] revealed how often the reason behind a specific touch style is difficult to pinpoint, with carers merely describing their touch-aversion as stemming from them not being ‘a touchy-feely person’. Lastly, [79] was the only study to highlight how expressive touch might be more amenable to personal adaptation, whereas instrumental touches are said to remain similar in terms of frequency and quality, no matter one’s own style and preferences.

Routasalo and Isola [86] and Edwards [76] also pinpoint how personal preferences might also be a strategy for emotional containment, mentioning how a nurse might decide to refrain from touching when they are embarrassed or to avoid excessive and perhaps uncontrollable displays of emotions.

The literature however also points us to the personal preferences of patients as a central element. Kelly et al [89] extend their narrative of ‘touch as a choice’ by arguing that, as much as nurses choose to touch, patients must be put in a position to be able to choose to be touched too. O'Lynn and Krautscheid [100] reported that patients feel powerless when they are not given the chance to express their touch preferences both before the engagement, and throughout it, even when they are aware that they are touched to be made comfortable and at ease. Usual touch preferences from the patient side often centre around wanting to be touched professionally (i.e., not too slow and lingering, but also not too fast, as if the practitioner is embarrassed), and being able to explicitly consent to most touch instances (see also: [78, 86]). This discussion around personal preferences also partly prefigures how patients seem to be able to read affective states and communicative elements from procedural touches, thus hinting at a certain permeability between the instrumental and expressive allowing patient-practitioner mutual understanding and attunement even in diagnostic manipulations. However, none of the studies highlighted here further described how the kinematics of instrumental touches create meaning.

De Luca et al. [83], Karlsson et al. [84], Estabrooks and Morse [90] and Gleeson and Higgins [99] all note that reading patients’ personal preference to touch is one of the central skills for a healthcare professional—done through observing open or closed body language, avoidant or welcoming eye contact, and sudden responses to touch (e.g., tilting away slightly).

### Theme IV: touch as a waltz

Within this theme, we clustered all studies that understood touch as a dynamically, ever-shifting and co-produced practice, adjusted at the moment based on bodily feedback and other contextual information. We borrowed the dance terminology from a previous review [39], as well as from some of the studies in this thread (e.g., [70]), which describe touch engagements in healthcare as a ‘silent, touching, moving dance’ (ibid.: 7). We find the metaphor particularly freighted with significance, because it encapsulates not only the conversational, embodied and interactional character of touch, as it has been cogently picked up by the studies below, but it also hints at the structural character of touch—in order to touch it is also necessary to be aware of the tacit rules of the dance one wants to engage in; or, in other words, medical touch is not just a conversation, but a conversation dictated and modulated by specific aims, rules and procedures.

We present the data as slotted across two sub-themes, one emphasising the relational and co-creative dimensions of touch, and the other attempting to tease out embodied, physical and adaptive practices of touch itself. We would like to emphasise how these two sub-themes cannot be disentangled from one another. Rather, these two dimensions of touch enable each other and are connected by, as well as rendering possible, the emergence of affective flows across touch dyads. In this sense, the two sub-themes speak not of simply co-occurring phenomena, but of co-constitutive ones. They are presented here as separate from one another only as an attempt to highlight their specific criticality while acknowledging that they are both constitutive and indispensable parts of the touch dance configured.

#### Touch as responsive co-creation

First, the dataset portion under exam understood touch as establishing an affective communicative space [68]: while this element has been reported also under the ‘Qualitative intents and meanings of touch’ theme, the studies in this cluster qualify this affective space opened up by touch as co-constructed, as well as stressing its malleable and adaptable nature. While previous literature argued that communicative touches happen at the start of a session, for instance with a handshake (e.g. [85]), only to then move to instrumental, medical touches, [70] describe the physiotherapy session itself as a conversation between bodies, where touch functions as a way to listen and attend to the other person’s needs.

By returning to phenomenology, both [70] and [89] thus argue that the caring touch is *pathic*, rather than *gnostic* [101, 102]: it is not merely an exercise in clinical judgment, gathering objective data from an inert body, but rather it is a bi-directional communicative process in which the body of the patient is alive and responsive. Consedine et al. [4] conclude from their observations of osteopaths at work that touch is an intricate and complex communicative process in which there is no subject and object, sender and receiver, patient and practitioner, agent and acted upon. This point is further strengthened by work with mental health nurses [103], which argues that emotions are exerted through touch, creating a link between body and mind, and between the body-minds of the two co-touchers: according to this, touch grounds the encounter by charging it with meaning and affect, and agency emerges as a networked property of these entangled bodies.

While the studies above focused on observations from the practitioners’ perspective, similar results also come from an interview study with patients undergoing chemotherapy [104]. Patients describe feeling gentleness, care and respect in the touch when the nurse accommodates patients’ tempo, because it signals to them that they are active participants in the engagement. They further described how, when the provider is solely focused on the task—acting in a ‘robotic manner’, as described by patients in another study [99]—, and excludes the patient as a co-participant, interactions become alienating, isolating and uncertain. They described for instance a patient who recounted the experience of being inserted into a nasogastric tube as unsettling, not so much for the procedure itself, which she had received before, but because she was deprived of her agency, i.e., her needs and desires were not met by the tactile engagements of the nurse, who just proceeded to insert the tube as they learnt from a book.

#### Touch as embodied praxis

By building on the previous sub-theme, what the literature in this sub-theme highlights is that for the above co-creative aspect to emerge, attention must be posited on *how* such dance is orchestrated. Touch within this narrative is thus understood as embodied, i.e. a tactile dance quickly adapting and responding to bodily feedback co-produced by the toucher and the touched. In this sense then, interaction requires holistic attention.

Bjorbækmo et al. [70] and Consedine et al. [4] for instance argue that the skin itself is the reactive epicentre of the engagement—with the osteopathic or physiotherapeutic session being a conversation between body and hands, requiring an inquisitive engagement where the patient’s body opens itself up to be gauged, and the hands of the practitioner are open to discovery and exploration while adapting their strokes and tempo based on the silent responses of the other. Both studies, as well as [90], recognise the difficulty in observing this adaptive dance: from the outside, this might just look like standard practice, but patients and practitioners alike describe it as an intuitive and instinctive conversation bursting from body and hands, a conversation which just ‘flows’ ([70]; see also: [4, 69]).

Bunzel et al. [72] highlights instead how reactions to touch could be grasped by facial expressions, but these are often overlooked by nurses since they usually stand behind or by the patient. They notice however how touch should not be seen as merely applied for medicine, and thus these reactions of the patient are ultimately what the touch should respond to: they then observed nurse dyads who shared agency while operating on a single patient—with one acting as the ‘eyes’ and the other as the ‘hands’.

In this context of touch as embodied and interactive practice, [91] ultimately understood touch as praxis—highly contingent and adaptable human action that transcends boundaries and transforms both actants in the process. Both [90] and [91] stress the importance of *cueing* in nursing as a way to gauge patients’ engagement in an incremental and dialogical way—establishing a rapport in which patients are not regarded as invalids amenable to medicalised intervention, but as complete and vibrant individuals open to enter into mutual pathic co-operation, as cogently explained by the interviewees in [104].

What the studies across these two sub-themes have in common is a renewed interest in human intentions and dialogical, co-creative adaptations, and how these cut through existing boundaries of patients and practitioners for instance, but also, and more crucially, that of instrumental and expressive touch, since ‘patients do not separate the perception of being touched into procedure-oriented touch versus touch intended to provide caring and comfort’ ([104]: 523; see also: [68]).

## Discussion

The review presented above draws an interesting picture regarding the use and communicative role of touch in allied medical professions, one which answers the questions set forth in the introduction. Summarily, the literature under scrutiny provided the following elements.

1. *What is communicated via touch?*
  Touch is understood as a nurturing presence signalling reassurance and empathy, and fostering communication and the formation of strong affective bonds. At the same time however, touch can be seen as a destabilising tool conveying control and coercion: explicit consent and verbalisation are thus often sought.
2. *Which touch instances tend to be seen as communicative?*
  While instrumental, procedural touches are described as being vastly more deployed, most studies highlighted a communicative value only in expressive, non-necessary touches. In this sense, while the latter are used to create an affective and haptic co-presence, the former are seen as mostly tools for medical and rehabilitative functions, unless part of procedures including supplemental expressive touches (i.e., ‘multiple intents touches’).
3. *What affects the use and communicative effectiveness of touch?*
  The communicative effectiveness of touch is described as greatly affected by the professional experience and role of the practitioner. Moreover, the gender of both practitioner and patient was seen as an important factor, e.g., patients enjoying the touch of male nurses less. Personal preferences, cultural factors and the emotional state of both the practitioner and the patient also were found to play a role – e.g., a nurse might refrain from touching when embarrassed.
4. *How is touch-mediated communication structured?*
  Touch was described a dynamic co-produced practice, with tactile engagements being adjusted in the moment based on bodily, affective and contextual feedback. Touch communication is thus seen as a bi-directional process in which the patient is an active co-participant. Such understanding also pushed against the conceptual boundaries between instrumental and expressive touch underlining most literature analysed.
5. *How have communicative elements of touch been picked up?*
  Most studies have focused on nursing – this might mean that communicative priorities from other fields might have been overlooked. Qualitative studies mostly used interviews and/or observational methods, but their integration and synergy seemed quite lacking. The most promising results regarding affect and communication were found in studies which deployed a phenomenological framework.

### Towards more conceptual variety

While the studies in the dataset highlighted important socio-communicative dimensions of medical touch, their limited contextual variety might have obscured a certain heterogeneity one could presumably expect, given the extensive remit of the field of ‘healthcare’. This is particularly cogent considering more than half of the studies summarised were conducted in one field (i.e., nursing), as well as the fact that only two studies analysed care scenarios in a non-EuroAmerican context.

As far as the first element is concerned, it is important to advocate for more research on touch in different healthcare professions. It is known [105, 106] that different professional contexts and disciplines have differing caring aims: while nurses tend to provide support by coordinating patient care within medical facilities, occupational therapists work with patients often in household contexts with the ultimate aim of stimulating and facilitating functional independence, whereas physiotherapists seek to promote greater range of mobility and confidence in movement mostly through prescribed exercises. This might mean that professionals will not merely perform different manipulations, as already highlighted by some studies in non-nursing contexts (e.g., [79]), but that these different caring aims might translate into differing communicative approaches to touch. In short, if our dataset, mostly comprised of studies in nursing, revealed that reassurance is the most communicated emotion, a renewed attention to touch in other disciplines might reveal that these practitioners tend to communicate different messages. Using occupational therapy as an example, a more thorough analysis of said profession might perhaps reveal that, given their rehabilitative approach towards functional independence, their touches might more often attempt to communicate feelings of security, or confidence (e.g., [69]).

An additional context on which much research has not been carried out is that of the relation between touch expression and efficacy, and the spatial context of the intervention. Within the dataset synthetised, only two studies [77, 80] mentioned how touch could be deployed more often in specific wards, or in the hospital as compared to home-based interventions. However, neither enquired into the reason as to why such changes might occur, as well as into how touch might be qualitatively different, rather than merely quantitatively, across domains.

Lastly, while the present review had specifically excluded touches performed *solely* by technological tools and/or autonomous agents, we maintained an openness towards touches that were *mediated* by tools and instruments. While such tools and mediated procedures abound in medical and nursing encounters (e.g., the use of stethoscopes, intravenous access via needles, and support with personal activities of daily living), very few of the studies under examination mentioned these. For example, [104] and [71] specifically describe the role of the tempo of execution of procedures such as needling and nasogastric intubation. However, even in such cases, no analytical attention is drawn in regard to the possible conceptual and somatic differences emerging from such human-through-instruments touches. On the one hand, such attention would have contributed to a more nuanced understanding of the complexity of haptic communication, as well as how tools influence the socio-cultural conceptualisation of ‘medical personhood’—something which has been already attempted in disciplines such as anthropology (e.g., [107, 108]). On the other hand, attention to such mediated encounters could have served as a preliminary step toward understanding the role and perception of technical tools. Such analytical attention will be fundamental to sketching out a larger-encompassing definition of the affective role of touch, one which can account for touches that might not be directly performed by humans (e.g., [109]).

Regarding cultural contexts, several studies already preliminarily outlined how touching style emerges through the interaction of different elements, including one’s upbringing and cultural background (e.g., [90, 96]). However, more work is necessary to better qualify these findings, exploring specific, localised sociocultural understandings of touch, and their direct impact on the use of touch within medical professions. In this sense, the work on proxemics spurred research into so-called ‘haptic behaviours’ [110, 111]—investigating different cultural attitudes towards personal space and bodily engagements, and how these impact the use of touch: [14] for instance argues how American culture is mostly ‘hands-off’, with a larger body bubble compared to Arabs, and recognising strangers violating that bubble as intruders, ‘causing the person to become defensive’ ([112]: 144). Other examples include work defensiveness (e.g., [113]), or culturally specific forms of relationship-building via social touch (e.g., [114]).

Such research agendas however never crossed over onto research in healthcare, so linking this knowledge to behaviours and attitudes in healthcare is only hypothesised and/or anecdotal. However, this movement would allow both a renewed appreciation for the role of culture even in medical settings, but also the establishment of an ethno-comparative perspective regarding touch in healthcare by exploring how medical practitioners in non-EuroAmerican contexts understand and deploy touch based on their own cultural presuppositions.

### Caring touch as a dance of intensities: addressing a crucial gap

The analysis of the dataset also foregrounded the strong division most studies seem to make between instrumental, task-related touches on the one hand, and expressive, affective touches on the other. Beyond merely categorising tactile engagements, this division functions to uphold borders between the communicative and social elements of touch, and its medical function. In doing so, medical procedures and manipulations are configured as devoid of meaning, beyond its diagnostic or relieving aims. When they have been said to function as forms of communication [69, 71], instrumental touches become so only in virtue of what we defined ‘additive touches’—extra tactile engagements that supplement the medical procedure, e.g. putting a hand on a patient’s shoulder while supporting their walk.

In this sense, nothing is said of the *quality* of the instrumental touch itself, and what kinds of communicative dimensions qualitative changes could provide to the touch. Do all nurses help a patient get up in the same way? What kind of difference does it make if one was to perform the manipulation slower, or faster? Or if their grip was lighter, or firmer? Could these be communicative and affective cues? While research in different contexts (e.g., [42, 115, 116]) shows that these modulations can produce specific affective responses, these findings have not been integrated into healthcare contexts, aside from brief mentions of some of these aspects (e.g., tempo modulation) in some of the literature examined in this review (i.e., [70, 104]).

Moreover, we had singled out a few studies which, by partly moving past the interest in empirically categorising, implicitly acknowledge a social component in all touches, including seemingly therapeutic procedures such as osteopathic palpitation ([70]; see also: [104]). These studies described the *embodied* and *instinctual* dimensions of tactile interactions, configuring touch not merely as a rational and intentional process that follows a linear path, but rather as tending towards the other’s needs by modulating bodily engagements accordingly. In this sense then, these studies refuse strong dichotomisations between the instrumental and the expressive: rather, all touch is seen as a primary example of *affect*. Within the humanities, the term has come to mean something different than in psychology, where it is mostly seen as a cognate for ‘emotion’. Instead, the recent ‘affective turn’ ([117]; see also [118, 119]), building upon the philosophies of Baruch Spinoza (e.g., [120]) and post-modernists Gilles Deleuze and Félix Guattari [121], understands affect as a pre-subjective, visceral force that influences (i.e., *affects*) our engagement with the world: the body in this sense is seen as a source of potentiality, and experience emerges as the transformative encounter between bodies, as modulation of these potentialities. When affect is understood as an exchange of *intensities* [122] between bodies, which in turn regulates bodies, invented boundaries between the ‘expressive’ and the ‘instrumental’ seem feebler, because affect cuts across them, as cogently argued by some of the studies in this dataset.

Consequently, a more robust engagement with these theories and cognates would allow a re-thinking of the theoretical scaffolding behind the study of touch in healthcare, recognising that all touch is affective in nature since it is predicated around an interactive engagement and exchange of intensities between two bodies, bodies which are attentive to one another via the medium of touch. In this sense, the studies which first hinted at the erosion of conceptual distinctions point us precisely to the fact that instrumental touches are never purely aseptic and devoid of affective meaning. Rather, touch is always a medium through which one modulates their engagement with an outside—and, in this case, another body—, and thus operates through constant re-negotiations and qualitative shifts which can respond to the needs of actants in the engagement. Touch is thus a reactive dance of intensities modulating perception and experience.

However, while we recognise the utmost centrality of reconfiguring touch by understanding the affective and relational nature of tactile engagements, we would also like to stress their simultaneous technical and rational nature. In this sense, some studies analysed (i.e., [70, 89]) borrow from phenomenology the distinction between the pathic and the gnostic, with the latter being cognitive, intellectual, and technical in a disembodied and de-contextualised manner, and the former being situated, relational, embodied and enactive. And while these studies constructed an either/or dynamics between the two, to ultimately argue that touch is pathic, we suggest that touch, *at least in healthcare*, should be seen as neither fully pathic nor fully gnostic. For instance, the procedures highlighted by the authors (e.g., support in rehabilitative exercises, osteopathic palpitations) are still caring-medical procedures, presumably learnt in a technical-diagnostic manner, only to then be adapted on the fly.

In this sense, this review has focused on studies on instrumental touch precisely to show the value of paying attention to the technical matrix of these touches, while also dealing with questions of affect and embodied knowledge. The analysis thus points to the need for future work that conceptually and empirically explores how touch in healthcare is distinct from many other instances of touch, precisely because of this complex intertwining between the gnostic and the pathic, the instrumental and the embodied. These conflictual elements seem to co-exist in what we could provisionally define as *caring touch*: a reactive dance of embodied intensities (the pathic), which has however medical, diagnostic and technical foundations (the gnostic). Let us reprise the metaphor of the dance which has guided both this analysis, but also the one of other reviews and studies (see, for instance: [39, 123]). Kelly et al. [39] for instance imagine the dynamics of touch as those of nineteenth-century Viennese ballroom, with dancers engaged in a classic Strauss waltz. Where we take issue is with how the metaphor has been used, or how it has been exemplified in empirical analyses, to downplay the importance of rules and knowledge to privilege the improvisational and dialogical. Saying that "some dance competently and yet look uncomfortable, […] clumsily follow[ing] the rules of the dance" (ibid.: 207) almost implies that the only thing that matters when engaging with one another is the "glid[ing] effortlessly in tune with the music and each other […] under the spell of the waltz" (ibid.: 207–208). While this might be true in some instances, it most likely is not when discussing touch in healthcare, where touch is never just an improvisational engagement, but is rather a matter of subtle, embodied, reactive, improvisational adjustments from a medical standard to engage more effectively and holistically with the other.

Starting from this premise, much empirical work is necessary to further explore how caring touch operates, particularly in terms of the qualitative shifts that are deployed for a given touch to deviate slightly from the medical standard in order to provide some form of non-medical, affective and communicative aim, as we will briefly sketch out in the following section.

Having said that, and before proceeding, a clarification must be made. The move made by this paper might seem confusing, and perhaps antithetical—particularly, anchoring our discussion in rather abstract and vaporous conceptualisations of affect after rooting most of our analysis in highly empirical work. What we are hinting at in this sense is a much wider-encompassing erosion of boundaries. Beyond leaving aside the dichotomisation between instrumental and expressive touch, we point to a need for erosion of disciplinary boundaries, boundaries which have implicitly created a division between conceptually adventurous writing on the touch that moves in similar directions to what we are suggesting here (see, for instance: [117, 124–132]), and much more grounded and objectivising work which seems somewhat reluctant to embrace critical insights coming from philosophy and the humanities. In this sense, we focused on the purpose of the latter approach, filtering out much interesting work that makes similar critiques to ours, to tease out many of the conceptual impasses emerging from such a myopic vision. We then, perhaps unintuitively, radically veer into different territories in this discussion precisely to stimulate and invite a generative encounter between these two strands which rarely cross one another.

### Dancing the dance of touch: towards a renewed attention to adaptive behaviours

Within this review and subsequent discussion, we have highlighted how some studies rightly attempted to move past the construction of dichotomous understandings of task-related touch and expressive, communicative ones, recognising instead that affect is present and mediates every embodied encounter. Simultaneously, however, we stressed the importance of not completely leaving behind the medical rationale of touch in healthcare. In this sense, the analysis provided points to one key question, of how this touch-dance actively and physically take shape in the form of adjustments, adaptations and qualitative changes to the instrumental touch operated by the medical professional to perform a medical task in a dialogical and communicative way. While some of these elements were summarily fleshed out by some studies under the rubric of ‘personal preferences’ [87], preferences and adaptations are not always aligned: while the idea of personal preference seemed to stress stylistic adaptations—i.e. different practitioners will touch and communicate differently—, adaptations here underline in-the-moment, reactive changes emerging from an encounter. For example, how would a physiotherapist modify their touch if they realise their patient is embarrassed?

The results and above discussion highlight that an exploration of these dynamics of touch encounters is of paramount importance, and research should strive to move beyond the conceptual level—which, as evidenced in the findings, focused on explaining *that* touch *is* dialogical, not so much on *how*—, in order to empirically describe how, while the touches performed by medical professionals are certainly not deliberate and are part of a whole set of protocolled manipulations and procedures, they are also often amenable to adaptations to engage with a patient and communicate something (e.g., security, reassurance, encouragement). Weiss [81] in this sense developed an instrument (i.e., the Tactile Interaction Index, TII) to record location, intensity, type of touch, and duration: these are interesting initial sensuous dimensions of touch, whose variation might tell us something about the communicative-affective instance. For example, what is a physiotherapist who is slowing down a passive range of motion exercise trying to convey? Or an occupational therapist who is providing hand-over-hand fine motor skills support, and does so by touching a smaller surface area of the hand? Or again, what about a nurse whose grip on a patient’s back while supporting their ambulation is firmer than usual? In this sense, recent research around the intersection between touch and affect (e.g., [42, 133, 134]) points us to an ever-increasing interest in the micro-dynamics of the touch experience—further complexifying the body into smaller and smaller units whose engagement carries different social and affective meanings. For example, a hand-supported grasping task might convey different feelings to the patient, based on which sub-region of the hand is being touched: touching the palm (hand-under-hand support) might increase their confidence and self-efficacy while touching the back of their hand (hand-over-hand support) might evoke feelings of constriction.

The present analysis and literature stress how these are important dimensions to address because they would also widen our conceptual understanding of the phenomenon. On the one hand, addressing qualitative changes and adaptations would mean acknowledging and embracing the temporality of touch. A tactile engagement is not something happening in a moment but rather extends in time: thus, it is not enough to simply enquire into the final communicative aim of one’s gesture. Rather, it is a question of exploring within (and beyond) the timescale of the touch when and how these shifts and adaptations are happening, what they mean from a communicative-medical perspective, and why and how they are implemented. On the other hand, empirically addressing this adaptive dimension of the dance of touch would allow us to better position and address the role of the two agents (i.e., the toucher-touched dyad) in this. In the field of human–computer interaction, work on meaning-making via tactile smart technologies [42] illustrated how touch is modulated alongside different axes (e.g., speed, heat, surface area, duration) to respond to different *affective scenarios*. An exploration alongside similar lines of enquiry could reveal how, similarly, healthcare professionals modulate and adapt different qualitative markers of their touch to respond to the arising of different emotional states in patients.

Lastly, in addition to such microscopic dimensions of the haptic engagement, affective and sensory flows also traverse a wider-encompassing social space that is not limited to the anatomical area affected by (or engaged in) the touch. Accounting for such enlarged spaces would allow for an understanding of embodied dynamics as operating across scales. Such scales might not necessarily encompass the strictly somatic, and might rather engage *with* the somatic in often difficult-to-trace ways. For instance, postural dynamics contributing to body language expression might be complicit in specific dialogical configurations of touch (or sometimes even lead to the choice of *not* touching) [135]; similarly, the presence of human spectators might play a role in how (and how much) touch is deployed [136], as could the specific spaces where touch is performed ([137, 138]; also hinted at in [77] and [80] in the review corpus). All these macroscopic interactional dimensions might contribute to the emergence of specific *affective atmospheres* [124] radically shaping the contours of the touch dance. While this line of enquiry has been brilliantly carried out in different contexts, from industrial robotics (e.g., [8, 139]) to Aikido martial arts (e.g., [140]), social studies of medicine and healthcare have been slower in integrating such insights.

### How to dance the dance of touch: notes on method for new conceptual horizons

It has been highlighted how recent discussions around touch in healthcare reorientate our attention to the affective value of instrumental touches themselves. Moreover, our last discussion point strongly advocated for using these insights to explore how said affective-communicative dimension takes shape in practice through adaptations of touch. In short, we highlighted the need for new conceptualisations around healthcare touch, allowing us to grasp the communicative value of all touches, irrespective of their assumed aim, as well as how much value emerges, is modulated, and changes via responsive re-adjustments in a dance-like manner.

However, empirically grasping such dialogical and reactive dimensions, while also capturing the implicit, subjective, and often difficult-to-pinpoint communicative aim behind it, might be challenging, as recognised for instance in [78].

While observation and detailed description of actual procedures seem to be the most fruitful way to get at this dimension, observation alone might not be sufficient, since these microscopic shifts in execution might be unnoticeable unless directly experienced and/or performed. In this sense then, few studies in the dataset combined observation and subsequent interviewing, but the modality of said mixed-methods approach moved in different directions, for instance by using observation to record and categorise instances of touch, and interviews to enquire around general aims and motivations behind touch. These studies thus presented very few actual descriptions of touch alongside the aim of said manipulation—either focusing on the former or discussing the latter in general. Following this, we suggest that further work in this field could rethink the connection between observation and interviewing, interlinking the two more strongly—i.e. observing specific manipulations, and the enquiring specifically about those via interviews. One study [69] accomplished said aim by videotaping tactile encounters between physiotherapist and patient, to then replay the tapes to the physiotherapist and gather their comments: quite tellingly, this study provided some of the richest descriptive insights into how healthcare professionals touch patients, and for what instrumental-communicative aims. Thus, videotaping sessions for subsequent discussion might be a possible way to stimulate conversation, as well as to support researchers in such a daunting task.

The use of video in qualitative research, and, in particular, well-established micro-interactional analytical approaches that draw from micro-ethnography (e.g., [141–143]) can afford a window into understanding the interactants’ *experiences* during the unfolding of interactive processes within a micro-scale [144]. Such details can provide insights into healthcare professionals’ and service users’ affective experiences from a phenomenological perspective as they interact with each other, respond to touch, or engage in, for example, modulating touch. Furthermore, an analytical approach that considers the whole-body's kinematics can go beyond a touch event, and incorporate the whole-body movement in the analysis, moment-to-moment. Such an approach can uncover the different ways in which touch occurs on the instrumental-affective continuum and the outcomes of touch. In this sense, micro-interactional approaches share conceptual overlap with conversation analysis methodologies that have been long employed to provide fine-grained descriptions of the temporal unfolding of semiotic interactions between agents. CA has been extensively deployed in medical contexts (see, for instance: [60, 61, 145–147]), but has rarely engaged with questions beyond linguistic content, as a review on the matter cogently points out [148]. A renewed attention to CA, particularly through the analytical and empirical attention towards embodied fine-grained interactions afforded by micro-interactional approaches might establish novel research areas in the study of touch as a communicative and affective resource.

Such re-orientations towards capturing the affective flows implicated in the sensuous and temporal unfolding of human interaction closely align with novel ethnographic dispositions. On the one hand, *sensory ethnography* [149, 150], is understood as an ethnographic sensibility towards the role sensuous engagements play in creating and understanding lived experience and meaning [151–153], might prove a fruitful avenue. A movement in this direction could produce thick descriptions of everyday engagements, revealing in turn how specific *haptic knowledge* [125] emerges in the specific encounters between practitioner and patient: some such work has been rapidly emerging in medical anthropology [154], albeit so far mostly from the perspective of haptic pedagogy.

On the other hand, relatedly, *multimodal ethnography* [155] might be a useful way forward to more robustly collect, catalogue and analyse haptic data in medical contexts. Given its strong methodological focus on dislocating the spoken/written as the central means to produce and represent knowledge, such an approach to data collection might present a more comprehensive and illustrative understanding of how touch contributes to meaning-making (see, for instance: [156–160]).

The use of bio-signals and sensors could also be an opportunity: while research using EEG revealed specific patterns of brain activation in the touched as the result of somatosensory stimulation via caressing touch [161], HCI studies using EMG hand- or arm-sensors on the toucher showed how muscle activity in the forearm and hands could also reveal aspects of affective engagements via touch (see, for instance: [162, 163]). Lastly, capacitive screens have been used to enquire into the force and intensity of touch, and the affective components of these engagement dimensions (e.g., [164, 165]). The use of such technologies for the collection of relevant data for a more fine-grained analysis of touch-mediated communication might however also open up under-investigated issues. For instance, participants’ emotional state might be compromised by being attached to a wearable device, no matter how unobtrusive that might be, particularly given the growing concern towards privacy and data sharing (e.g., [166]). In this sense, further work that enquires into such possibilities by using such devices to explore both their efficacy, but also the impact they have in potentially disrupting the naturalness of non-verbal communication between humans, is necessary.

It has also been noted that the studies that started to tease out this reconceptualization of healthcare touch mostly grounded themselves within phenomenological theory. In this sense, phenomenology seems to be an apt lens to analyse the topic, since it allows a re-appraisal of the role of the body in knowledge acquisition and action. However, the studies considered mostly used phenomenology as *theory* to design the data collection and to parse through the data, rather than as *method* itself. As noted above, what we are left with are then certainly illuminating conceptual considerations, but very few practical examples and detailed descriptions of the phenomenon as it happens. In this sense then, the connection between observation and retelling via interview mentioned above might be strengthened also by using theoretical insights from phenomenology as methodological orientation. For example, work around *micro-phenomenology* (e.g., [167–170]) as interviewing technique suggests how a renewed attention to gestures, actions and associated feelings *at the moment* opens up a more fine-grained entry point into lived experience and embodied interaction. Such an approach within the field of healthcare communication might thus reveal more precisely all the elements that have been partly obscured in the literature analysed, and which we have highlighted in this discussion.

### Limitations

While the present review has been conducted as rigorously as possible, some evident limitations might have partly shaped the results, and following discussion.

Firstly, the study was conducted throughout 2022, at the tail-end of the COVID-19 health emergency. The pandemic particularly hit healthcare fields, and, more crucially, it drastically reshaped the ways in which professionals are allowed to physically engage with patients (e.g., [171–176]). We expect upcoming studies to discuss more robust questions around touch and lack thereof in care environments.

Secondly, the dataset sampled only included studies written in English, French, Italian, Spanish and Finnish. This was done out of convenience since these are the only languages fluently spoken by the review team. However, throughout the discussion, it has been noted that the literature under scrutiny under-addressed questions around cultural specificity of touch, evidenced by the lack of studies carried out in non-EuroAmerican contexts. It is entirely possible that said studies exist but were not available in the languages selected (see, for example: [177]).

Thirdly, the analysed corpus can be considered somewhat small, but still in line with the average number in similar reviews (i.e., [31, 34, 36, 38, 39]). This emerged as a result of the inclusion criteria used, which allowed us to focus solely on highly empirical work carried out in decidedly medical contexts. In so doing however, much interesting work which attempts to conceptualise touch in novel, and often far-reaching, ways had to be excluded (see, for example: [117, 126–128, 178]). Some of this work, and the much-needed conceptual variety it brings, has been re-injected into the manuscript in the discussion, precisely to critique the findings and demonstrate how much has been done to conceptualise touch in adjacent disciplines. Our intent in focusing on a relatively modest subset of work on touch was to highlight a somewhat prevalent analytical lens emerging in very applied, evidence-based healthcare studies, in order to subsequently highlight what such a vision might obscure.

Lastly, as noted in the findings, 58% of the studies included in this synthesis focused solely on practitioners’ attitudes towards touch, making our analysis skewed towards a singular perspective. This is a grave limitation when considering the dyadic and dialogic nature of touch highlighted in the discussion, made even more glaring by the recent calls in healthcare forums regarding widening stakeholders’ inclusion (see, for example [179, 180]). While our search specifically included the term ‘patient’, such an omission might be attributable to a lack of sensitivity to the matter in our search strategy; more cogently and more plausibly, it might be symptomatic of a general lack of interest in understanding touch from the perspective of patients, speaking to the need for further research including all relevant stakeholders towards wider-encompassing evidence-based practice.

## Conclusion

Within this review, we set to explore how naturalistic studies within healthcare settings have described the touch used by professionals when tending to patients. Particularly, we have focused on the affective value and communicative aims that touch can assume in these contexts. Through the analysis of 36 studies, we discovered that most work within the field abides by a division between instrumental, task-related touches, and non-necessary, affective touches, with the former being overwhelmingly more often deployed, but with the latter carrying most, if not all, communicative salience. Touch was found to be a strong positive facilitator to the establishment of social bonds between patient and practitioner; at the same time, its unregulated use could be perceived as controlling.

By taking into consideration a smaller sub-set of studies which focused on medical touch from an embodied perspective, we further highlighted this interactional and reactive dimension of touch by stressing how affect could be seen as a central part of every form of touch. Thus, we encouraged more work in the field, in order to both rethink healthcare touch conceptually and to better understand how even instrumental touches might have a communicative function. This function might be grasped by observing subtle, and often difficult to capture from the outside, shifts in execution responding to the physical and affective needs of the patient. We suggested that such rethinking happen both at the conceptual level and at the methodological level, experimenting with tools and methods which could allow us to get closer to the embodied, lived experience of both the toucher and the touched.

This review has profound implications for social studies of non-verbal communication in healthcare. On the one hand, we have highlighted a crucial gap in the literature by attempting to reconcile two approaches by highlighting affect as a driver of all touch, while also not leaving aside the fact that we are dealing with medical procedures. This attention to both the instrumental *and* the communicative facets of medical touch could, in turn, inform conceptualisations of touch outside of the social sciences, for instance by (1) contributing to the development of embodied technologies that could support patients via touch; (2) providing pragmatic guidelines for practitioners regarding how to understand and carry out diagnostic and instrumental procedures while also paying attention to their social and affective valence; (3) design teaching curricula which emphasise the human dimension of medical procedures, training healthcare professionals towards more attention to the patient as an active agent in the process of providing medical treatment.

## Appendix 1

**Table:** ENTREQ Checklist

Item	Reported on page
Aim	5, 16–17
Synthesis methodology	7–8
Approach to searching	5
Inclusion criteria	5–7, Table 1
Data sources	5
Electronic search strategy	Supplementary file ‘Database searches’
Screening methods	5, Fig. 1
Study characteristics	14, Table 2
Study selection results	6–7, Fig. 1
Rationale for appraisal	5–6
Appraisal items	6
Appraisal process	6–7
Appraisal results	7
Data extraction	7
Software	7
Number of reviewers	5–7
Coding	7
Study comparison	7, 9, 14
Derivation of themes	7, 9, Appendix 4
Quotations	Appendix 4
Synthesis output	14–22, Appendix 4, Appendix 5

## Appendix 2

**Table:** PRISMA 2020 Checklist

Section and Topic	Item #	Checklist item	Location where item is reported
**TITLE**			
Title	1	Identify the report as a systematic review	1–3
**ABSTRACT**			
Abstract	2	See the PRISMA 2020 for Abstracts checklist	2
**INTRODUCTION**			
Rationale	3	Describe the rationale for the review in the context of existing knowledge	3–5
Objectives	4	Provide an explicit statement of the objective(s) or question(s) the review addresses	5
**METHODS**			
Eligibility criteria	5	Specify the inclusion and exclusion criteria for the review and how studies were grouped for the syntheses	6–7, 14, Appendix 4
Information sources	6	Specify all databases, registers, websites, organisations, reference lists and other sources searched or consulted to identify studies. Specify the date when each source was last searched or consulted	5
Search strategy	7	Present the full search strategies for all databases, registers and websites, including any filters and limits used	Supplementary file “Database searches”
Selection process	8	Specify the methods used to decide whether a study met the inclusion criteria of the review, including how many reviewers screened each record and each report retrieved, whether they worked independently, and if applicable, details of automation tools used in the process	6–7
Data collection process	9	Specify the methods used to collect data from reports, including how many reviewers collected data from each report, whether they worked independently, any processes for obtaining or confirming data from study investigators, and if applicable, details of automation tools used in the process	7–8
Data items	10a	List and define all outcomes for which data were sought. Specify whether all results that were compatible with each outcome domain in each study were sought (e.g. for all measures, time points, analyses), and if not, the methods used to decide which results to collect	N/A
	10b	List and define all other variables for which data were sought (e.g. participant and intervention characteristics, funding sources). Describe any assumptions made about any missing or unclear information	6
Study risk of bias assessment	11	Specify the methods used to assess risk of bias in the included studies, including details of the tool(s) used, how many reviewers assessed each study and whether they worked independently, and if applicable, details of automation tools used in the process	6–7
Effect measures	12	Specify for each outcome the effect measure(s) (e.g. risk ratio, mean difference) used in the synthesis or presentation of results	N/A
Synthesis methods	13a	Describe the processes used to decide which studies were eligible for each synthesis (e.g. tabulating the study intervention characteristics and comparing against the planned groups for each synthesis (item #5))	6–7
	13b	Describe any methods required to prepare the data for presentation or synthesis, such as handling of missing summary statistics, or data conversions	N/A
	13c	Describe any methods used to tabulate or visually display results of individual studies and syntheses	7
	13d	Describe any methods used to synthesize results and provide a rationale for the choice(s). If meta-analysis was performed, describe the model(s), method(s) to identify the presence and extent of statistical heterogeneity, and software package(s) used	7–8
	13e	Describe any methods used to explore possible causes of heterogeneity among study results (e.g. subgroup analysis, meta-regression)	8
	13f	Describe any sensitivity analyses conducted to assess robustness of the synthesized results	N/A
Reporting bias assessment	14	Describe any methods used to assess risk of bias due to missing results in a synthesis (arising from reporting biases)	N/A
Certainty assessment	15	Describe any methods used to assess certainty (or confidence) in the body of evidence for an outcome	8
**RESULTS**			
Study selection	16a	Describe the results of the search and selection process, from the number of records identified in the search to the number of studies included in the review, ideally using a flow diagram	5–8; Fig. 1
	16b	Cite studies that might appear to meet the inclusion criteria, but which were excluded, and explain why they were excluded	7
Study characteristics	17	Cite each included study and present its characteristics	Table 2
Risk of bias in studies	18	Present assessments of risk of bias for each included study	Appendix 6
Results of individual studies	19	For all outcomes, present, for each study: (a) summary statistics for each group (where appropriate) and (b) an effect estimate and its precision (e.g. confidence/credible interval), ideally using structured tables or plots	Appendix 5
Results of syntheses	20a	For each synthesis, briefly summarise the characteristics and risk of bias among contributing studies	Table 2, Appendix 6
	20b	Present results of all statistical syntheses conducted. If meta-analysis was done, present for each the summary estimate and its precision (e.g. confidence/credible interval) and measures of statistical heterogeneity. If comparing groups, describe the direction of the effect	Appendix 4, Appendix 5
	20c	Present results of all investigations of possible causes of heterogeneity among study results	Appendix 5
	20d	Present results of all sensitivity analyses conducted to assess the robustness of the synthesized results	N/A
Reporting biases	21	Present assessments of risk of bias due to missing results (arising from reporting biases) for each synthesis assessed	N/A
Certainty of evidence	22	Present assessments of certainty (or confidence) in the body of evidence for each outcome assessed	Appendix 5
**DISCUSSION**			
Discussion	23a	Provide a general interpretation of the results in the context of other evidence	22–28
	23b	Discuss any limitations of the evidence included in the review	29
	23c	Discuss any limitations of the review processes used	29–30
	23d	Discuss implications of the results for practice, policy, and future research	29–30
**OTHER INFORMATION**			
Registration and protocol	24a	Provide registration information for the review, including register name and registration number, or state that the review was not registered	Supplemental document
	24b	Indicate where the review protocol can be accessed, or state that a protocol was not prepared	Supplemental document
	24c	Describe and explain any amendments to information provided at registration or in the protocol	Supplemental document
Support	25	Describe sources of financial or non-financial support for the review, and the role of the funders or sponsors in the review	Supplemental document
Competing interests	26	Declare any competing interests of review authors	Supplemental document
Availability of data, code and other materials	27	Report which of the following are publicly available and where they can be found: template data collection forms; data extracted from included studies; data used for all analyses; analytic code; any other materials used in the review	Supplemental document

## Appendix 3

**Table:** PRISMA 2020 Abstract Checklist

Section and Topic	Item #	Checklist item	Reported (Yes/No)
**TITLE**			
Title	1	Identify the report as a systematic review	YES
**BACKGROUND**			
Objectives	2	Provide an explicit statement of the main objective(s) or question(s) the review addresses	YES
**METHODS**			
Eligibility criteria	3	Specify the inclusion and exclusion criteria for the review	YES
Information sources	4	Specify the information sources (e.g. databases, registers) used to identify studies and the date when each was last searched	YES
Risk of bias	5	Specify the methods used to assess risk of bias in the included studies	YES
Synthesis of results	6	Specify the methods used to present and synthesise results	YES
**RESULTS**			
Included studies	7	Give the total number of included studies and participants and summarise relevant characteristics of studies	YES
Synthesis of results	8	Present results for main outcomes, preferably indicating the number of included studies and participants for each. If meta-analysis was done, report the summary estimate and confidence/credible interval. If comparing groups, indicate the direction of the effect (i.e. which group is favoured)	YES
**DISCUSSION**			
Limitations of evidence	9	Provide a brief summary of the limitations of the evidence included in the review (e.g. study risk of bias, inconsistency and imprecision)	YES
Interpretation	10	Provide a general interpretation of the results and important implications	YES
**OTHER**			
Funding	11	Specify the primary source of funding for the review	YES
Registration	12	Provide the register name and registration number	NO

## Appendix 4

**Table:** Findings and themes allocation report

Authors	Findings	Descriptive themes	Analytical themes	Quotations
Adomat and Killingworth (1994) [25]	- No significant relationship between length of service and instrumental touch - Nurses with less than 2 years of experience engage in significantly more caring touch - No significant relation between nurses’ age and touch type	- Touch and experience - Touch and age	III. Touch and its actors	No quotes provided
Barnett (1972) [23, 77]	- Registered nurses touched the most, while interns do not touch patients (hypothesised it is because they are trying to achieve role expectations) - The younger the medical personnel, the more touch - Female staff touches 85% more than male staff - Hands, forehead and abdomen are the most touched areas - Paediatrics and ITUs are the wards where touch is used the most	- Touch on the body - Touch and gender - Touch and caring context - Touch and age - Touch and experience	I. Quantifying touch III. Touch and its actors	N/A (quantitative)
Bjorkbækmo and Mengshoel (2016) [70]	- Touch perception is embodied: an observant perceives tempo differently - PTs quickly respond to patients’ bodily reactions, adapting strokes and tempo to patients’ needs - Touch is a dialogue, with both parties engaged in non-verbal communication - Touch opens up affective and communicative spaces where learning happens through non-verbal exploration and interaction - Touch in physiotherapy is pathic	- Touch as embodied interaction - Touch as response - Touch and tempo	IV. Touch as waltz	“It is not necessary [to verbalise preference] […] he understands|. I don’t have to tell him. […] Maybe he can see it on my face” (6)
Bundgaard et al. (2011) [71]	- The amount of touch contact is dictated by individual preferences of both nurse and patient, and context - Too much touch can be perceived as controlling - Exchange of caring touch is often supplementary (e.g. hand on shoulder during endoscopy) - Touch can create a sense of presence, enhancing the feeling of being cared for	- Touch as risk - Multiple intents of touch - Touch as humanising presence - Touch as preference - Touch as communication and care	II. Qualitative intents and meanings of touch IV. Touch as waltz	“Yes, this contact makes me feel safe, [because] I feel that someone is present” (36) “Their touch reveals if they are present or not. I very soon can detect if they are actually here for me in this situation” (36) “Sometimes, if they are anxious, I feel it in their handshakes” (36)
Bunzel et al. (2020) [72]	- Facial expressions are very important to understand people’s reactions to touch - In some scenarios nurses cannot see this because of how they are positioned, or because they are too focused on the practical task - Touch has to be dependent on the patient’s response – it is thus vital to always be mindful of the patient’s reactions	- Touch as response - Touch, vision and collaboration	IV. Touch as waltz	“I did see the grimacing, but I didn’t really notice what it was about.” (951)
Caris-Verhallen et al. (1999) [80]	- Affective touch recorded in more than 40% of nursing encounters - It however amounts to only 1–5% of total encounter time - Touch can be used to reaffirm verbalised empathy: verbal and non-verbal communication are intertwined - Nurses in care homes displayed a more affective touch - Affective touch is highly dependent on the nurses’ style	- Instrumental vs affective touch - Touch and caring context - Touch style - Touch as communication and care	I. Quantitatively mapping touch II. Qualitative intents and meanings of touch III. Touch and its actors	N/A (quantitative)
Cocksedge et al. (2013) [85]	- Communication is central in doctor-patient relationship, since it helps in building a relationship of care - Using touch improves communication quality, showing an empathetic interest in patients - Being touched made patients feel understood and reassured - GPs clearly distinguished between expressive and procedural touch, but also mentioned that the two can coexist – procedural touch can be caring in itself - Age, sex, and bereavement situations were factors influencing the use of touch: bereaved and older patients were touched the most; male GPs’ touch was felt as embarrassing and weird, especially when touching a woman	- Touch and gender - Touch and age - Touch as communication and care - Multiple intents of touch	II. Qualitative intents and meanings of touch III. Touch and its actors	“[Being touched made me feel] that they understood.” (285) “You’re performing a practical task for them to help them, other than just trying to comfort them. And, although it may be helpful, you know, in a way it’s reassuring to them.” (286) “I think I would be more cautious if it was a woman […]. I wouldn’t really want to be put in the situation of being accused of touching a woman” (287)
Consedine et al. (2016) [4]	- Touch in osteopathic sessions involves intricate and complex communicative interactions. Touch is thus a dialogue where the patient is simultaneously object and a subject - Inquisitive engagement: the patient’s body is open to be gauged, while the osteopath’s touch is open to discovery and exploration - The body (of patient) and the hands (of the osteopath) are in a conversation in which judgements and questioning happen non-verbally—e.g. hands function as a way to ask “is this stiff? Does it feel easy to manipulate? How does it feel?” - Touch can make patients feel nurtured and supported - Care is mediated via touch (tactile care) - Tactile care is responsive and bi-directional, rather than prescriptive and procedural	- Touch as embodied interaction - Touch as response - Touch as communication and care	II. Qualitative intents and meanings of touch IV. Touch as waltz	“So you are in dialogue with touch, it is definitely part of the deal because he is feeling and making interpretations” (7) “I guess it is the same sort of touch that you get when you are in hospital […]. You sort of feel relaxed and comfortable and nurtured” (7) “[Touch] just gives you that kind of feeling of he knows where you have been or what your body has been going through. It does make you feel cared for” (7) “But the other thing that struck me as quite extraordinary is that he seems to see with his fingers.” (8)
De Carvalho de Rezende et al. (2015) [67]	- Light touch is more present among nursing staff - Indifferent touch is involved in technical procedures, particularly by nurses - Touch is seen as essential to nursing care because it adds a humanising dimension to nursing - Nurses touch more than doctors	- Light and indifferent touch - Touch as communication and care - Touch and experience	I. Quantitatively mapping touch II. Qualitative intents and meanings of touch	N/A (quantitative)
De Luca et al. (2021) [91]	- Touch is often desired because it provides mutual pleasure, facilitating the establishment of a bond - It is still however a preference, and practitioners need to know that the same type of touch dialogue might not work with all patients - Touch was seen as a natural and in-built dimension of human nature and sociality - Touching is seen as embodied action: it’s highly contingent and adaptable (e.g. drawing blood via tourniquet can be done while keeping an arm on the patient’s shoulder, to signify closeness)	- Touch as embodied interaction - Touch as humanising presence - Touch as communication and care - Touch as preference	II. Qualitative intents and meanings of touch III. Touch and its actors IV. Touch as waltz	“[Touch] helps to communicate with the patient, make it easier, reinforcing nonverbal messages we want to pass” (4) “[Sometimes] you see that they smile at you [when touched], or you see that they look at you not wanting any closeness with you” (5) “I realised how important it is and I see this when I draw blood, when I put the tourniquet on. Before I didn’t use to leave any hand on the patient, now I try to maintain contact with their arm and I keep it there till the end until I remove the tourniquet” (6)
De Luca et al. (2022) [83]	- Touch is a resource since it is simultaneously a technical tool to acquire knowledge, but it also provides procedural care, physical comfort and emotional support - Nurses need to be able to read people’s preferences, for instance through body language (open/close), or eye contact	- Touch as preference - Multiple intents of touch - Touch as response	II. Qualitative intents and meanings of touch III. Touch and its actors	“[during touch] I can get a gauge from them if they make eye contact with me. If their body language is open or closed.” (460) “In the hospital touch was pretty much continuous and I do recall trying to capture opportunities to be more therapeutic or intentional.” (460)
Dobson et al. (2004) [87]	- Touch is based on personal preference – some people (both practitioner and patient) just do not enjoy touching beyond the strict necessary - Some practitioners are not ‘touchy feely’—it’s their style - Touch can unconsciously become a tool of communication, providing care and reassurance - Touch has sensory specifications: gradation, type, style, sensitivity all affect the experience of the touch - Touch is always interpreted by the touched. Carers need to be aware of this at all times (empathetic reflection)	- Touch as preference - Touch as communication and care - Touch style	II. Qualitative intents and meanings of touch III. Touch and its actors	“Everybody’s response to touch is different” (121)
Eber (2018) [103]	- Agency (of both practitioners and children) emerges through a network of bodies connected via touch: touch gives meaning to a relationship by grounding it in physicality - Emotions can be exerted through touch. Mind and body are thus connected via the medium of touch (embodiment thesis)	- Touch as embodied interaction	IV. Touch as waltz	N/A (ethnographic)
Edwards (1998) [76]	- Hands, arms and lower legs are seen as safe zones for touching - Nurses have to ensure that patients are comfortable with the touch - Nurses also have emotions: they might get embarrassed, and thus reduce touch (e.g. ask patient to wash their genitalia themselves) - Touch can also work as a means of persuasion - Patients are expected to be touched by nurses, but not the reverse	- Touch as preference - Touch on the body - Touch as persuasion - Directionality of touch	I. Quantitatively mapping touch III. Touch and its actors	N/A (ethnographic)
Estabrooks and Morse (1992) [90]	- Participants could not really define touch. Rather, it is seen as a multi-dimensional gestalt that includes but is not limited to, tactile contact. Posture, voice tone, affect, intent and meaning are all part of what one sees as ‘touch’ - Some of these elements are graspable via observation, but others are not, making the study of touch from a third-view perspective difficult - Learning how to touch occurs in three stages: 1. Cultural background; 2. Nursing school; 3. Practice - Cues and cueing are vital to effective touch: a nurse needs to be able to understand patients’ reactions through bodily cues, in order to understand the appropriateness of their touch intervention	- Touch as preference - Touch as response - Touch style - Touch and experience	III. Touch and its actors IV. Touch as waltz	No quotes provided
Gale and Hegarty (2000) [78]	- Of 193 instances of touch, 55% were seen as functional, 25% as expressive - Hands, head, trunk, and arms are the most touched parts - Practitioners gather information regarding patients’ reactions through facial expressions and eye contact - Expressive touch seemed to be emerging only after nurses had established a rapport with the client	- Touch on the body - Touch as response - Touch and time	I. Quantitatively mapping touch III. Touch and its actors	N/A (quantitative)
Gleeson and Higgins (2009) [99]	- Expressive touch is seen as a powerful tool, used to reassure, comfort and break barriers between nurse and client - However, instrumental touch should also not be ‘robotic’, otherwise the experience for the patient would be negative - Touch needs to be tailored to clients’ and nurses’ preferences – the effectiveness of such tailoring is assessed through non-verbal and verbal cueing - Touch can connect on an emotional level, particularly when verbal communication is seen as inadequate or not possible - It is challenging for men to touch women, because of possible allegations of sexual impropriety. Men discussed using touch more sporadically and cautiously when treating female patients	- Touch and gender - Touch as communication and care - Touch as response - Touch as preference - Touch as risk	II. Qualitative intents and meanings of touch III. Touch and its actors IV. Touch as waltz	“[Touch] seems to comfort them […] and gives them a better feeling of security” (385) “You have to be very wary of your behaviour and especially with touch. There is a heightened sensitivity […] [touch] can come across as quite threatening” (385) “Maybe the verbal reassurance isn’t just enough, so just to add a little bit more, there’s the actual reassurance of touch as well” (386) “I would be wary, as a male, of young female, certainly. It’s about protecting yourself from any allegations…” (387)
Hollinger and Buschmann (1993) [181]	- Residents and registered nurses perceived nonprocedural touch as more positive - Licensed nurses perceived procedural touch as more positive - Residents and caregivers perceived touch above the waist as more positive - Overall, procedural touch seems to be preferred	- Touch on the body	I. Quantitatively mapping touch III. Touch and its actors	N/A (quantitative)
Jung and Fouts (2011) [66]	- All types of caregivers provide a more passive social-affectionate touch - Gender did not play a significant role	- Touch and gender	I. Quantitatively mapping touch III. Touch and its actors	N/A (quantitative)
Karlsson et al. (2022) [84]	- Emotional touch opens up a conversational space that produces empathy: safety, reassurance, calming - Caring touch is both emotional and imperative: it is imperative because it provides factual knowledge; it is emotional because it enhances the caring relationship - In order for touch to be caring, it needs to be conversant and adaptable - Reading body language to understand appropriateness	- Touch as communication and care - Multiple intents of touch - Touch as response	II. Qualitative intents and meanings of touch III. Touch and its actors	“I am not that person that touches everyone, I try to read their body language, to reassure that the touch isn’t inappropriate” (3) “If I have to touch a patient who does not like to be touched, I only do what is required within the nursing care” (3) “The patient will flinch if he or she does not like to be touched. If so, you have to respect that because not all people like to be touched” (4) “A lot of patients in the ICU are severely ill, therefore I believe that […] you need a lot of closeness. I can give comfort by being close.” (5) “The patient connected the voice to a person who hadn’t been adaptable in the way she touched while caring” (4)
Kelly et al. (2019) [89]	- Choosing how to touch is a personal choice, influenced by cultural background - Patients must also choose to be touched: doctors need strategies to invite touch, and make patients feel comfortable (e.g. shaking hands at the beginning of session) - Touch can also be used as a response to patients’ distress (e.g. when breaking a bad news) - Touch in healthcare is seen as pathic, embodied and relational	- Touch style - Touch as preference - Touch as embodied interaction - Touch as communication and care	II. Qualitative intents and meanings of touch III. Touch and its actors IV. Touch as waltz	“I think I’m empathetic but I don’t necessarily feel the need to, so I guess I don’t use touch as communication” (403) “Both the patient’s culture and the physician’s culture [influence touch].” (403) “I think touch really probably forms the basis of the relationship that one might have with your patients, because it can be a very positive experience or it can be a very negative experience depending on the quality of the touch” (403) “We share many intimate and deeply personal moments […] touch is a big part of what we do in the consultation and breaking news of all kinds” (404)
Kelly et al. (2020) [96]	- Touch as presence: touch acknowledges patients’ vulnerability, showing practitioners’ presence and reassuring abilities, as well as compassion (e.g. holding hands with an anxious patient) - Touch is also risk: it is experienced differently based on age, gender, ethnicity and cultural background, and can be misinterpreted - Touch thus needs to be dynamically and dialogically reconfigured	- Touch as risk - Touch and gender - Touch as humanising presence - Touch as response	II. Qualitative intents and meanings of touch III. Touch and its actors IV. Touch as waltz	“The act of touching another person is to remind yourself that you are human and that they are human and that we are connecting” (1895) “We need to be careful [when touching]” (1896)
Leonard and Kalman (2015) [104]	- Patients want to be regarded as themselves, as complete and vibrant humans, not as invalids. Touch can support or hinder this because it can support the establishment of a rapport that transcends medical boundaries (expert vs invalid). Willingness to establish such a rapport is necessary; otherwise, touch will be impersonal and medicalised - For example, gentle touches when providing IV access communicate a feeling of respect and sensitivity. Similarly, accommodating patients’ tempo can foster a relationship of mutual respect and trust - When the provider is solely focused on the task and excludes the patient as a co-participant, interactions are alienating, leading to isolation and uncertainty	- Touch as embodied interaction - Touch as response - Touch as communication and caring - Touch and tempo - Multiple intents of touch	II. Qualitative intents and meanings of touch IV. Touch as waltz	“The varieties [of touch] I would say […] were from fabulously compassionate and […] great to […] just very detached and just doing their j-o-b job – not participating in you” (519) “Rather than coming at you all fast and in a hurry […] it’s a soft, caring touch” (519) “[The physician doing the colonoscopy] had to do a little exam, and he asked permission […], told me what he was doing […], what was going on. His hands were very soft, very gentle, [and] nothing hurt. He’s […] very kind, very dedicated […]. He was very respectful.” (519)
McCann and McKenna (1993) [20]	- Of 149 touches recorded, 142 were seen as instrumental, and 7 as expressive - Arms, legs, shoulders, back and hands are the most touched body parts - Patients stated they would feel uncomfortable if touched expressively by a male nurse	- Touch on the body - Instrumental vs affective touch - Touch and gender	I. Quantitatively mapping touch III. Touch and its actors	No quotes provided
Mononen (2019) [68]	- Stroking and embracing are the most typical means to realise affective touch - Boundaries between affective and assistive touch are fuzzy - Affective touch regulates participation: touch is used to gather attention to a task by establishing a participation framework, orientating both patient and carer towards a task. A stroke can then for example be used to maintain and foster affect, because redirecting the patient through verbal reinforcement - Touch thus signals haptic co-presence within a shared social space	- Multiple intents of touch - Touch as embodied interaction - Touch as humanising presence - Touch as persuasion - Modalities of touch	II. Qualitative intents and meanings of touch IV. Touch as waltz	N/A (ethnographic)
Morris et al. (2014) [79]	- 80% of recorded touches were instrumental - On average, 9.8 instrumental touches each session; 2.5 expressive touches - 43% of instrumental touches related to functional mobility; 24% to provide instructions, and 17% to adjust equipment - Female practitioners used expressive touch twice as often as male practitioners - Male practitioners used instrumental touch 33% more often - Use of expressive touch seemed based on individual style, whereas instrumental touch was not	- Touch style - Instrumental vs affective touch - Touch and gender	I. Quantitatively mapping touch III. Touch and its actors	N/A (quantitative)
Mulaik et al. (1991) [98]	- Patients viewed touch as demonstrating care and affection - Some others also believed it conveyed control, and thus it should be used sparingly - 74% of respondents mentioned nurses’ touch feels good - Instrumental touch is more deployed than optional touch - Younger nurses tend to touch more and more often - Men received more optional touch than women	- Touch as risk - Touch as communication and care - Touch and gender - Instrumental vs affective touch - Touch and age	I. Quantitatively mapping touch II. Qualitative intents and meanings of touch III. Touch and its actors	N/A (quantitative)
O’Lynn and Krautscheid (2011) [100]	- Participants feel powerless when they’re not given the chance to express their touch preferences, or their preferences are not accommodated - Most of the younger female participants said they’d prefer being touched by a woman - Men similarly preferred being touched intimately by a female practitioner - Some men mentioned sexual orientation: being touched by a gay nurse might feel embarrassing - Participants wanted to be touched professionally: speed seems to play a role in professionality, as being touched too fast would feel like the nurse is embarrassed and wants to rush through the manipulation; a slow tempo on the other hand would feel creepy and disrespectful	- Touch as risk - Touch and tempo - Touch and gender - Touch as preference - Touch and sexual orientation	II. Qualitative intents and meanings of touch III. Touch and its actors	“Any kind of hesitancy [i.e. slow tempo] would make me feel more anxious and less inclined to let [a nurse touch me]” (26) “Let me make the decision! [i.e. regarding how to be touched]” (28) “If it could happen that I could have someone of the same gender, that might make me more at ease” (28) “Too fast almost seems like they are trying to avoid the situation […] [but] linger[ing] too long in one area [would be] creepy, or make the person feel disrespected” (29)
Pedrazza et al. (2018) [182]	- Nurses present high levels of comfort with task-oriented touch, whereas they feel less at ease providing support and containment through touch - Task-oriented touch was found to be related to attachment insecurity and worry: insecure caregivers are more likely to experience difficulties - Strong association between worry and touch promoting comfort. Nurses might be worried because the said benefits of touch are outside standard protocol	- Touch as preference	III. Touch and its actors	N/A (quantitative)
Pratt and Mason (1984) [88]	- 4 or more intentions behind touch were used to describe most situations - Communicative intentions are seen in most instances: participants sub-divided such categories into ‘reassuring’, ‘comforting’, ‘providing security and ‘restraining’ - Few touches were seen as only instrumental - The ‘communicative’ touch category was the one where participants provided the most additions	- Multiple intents of touch - Touch as communication and care	II. Qualitative intents and meanings of touch	N/A (quantitative)
Roger et al. (2002) [69]	- Clinical experience gives physiotherapists increased knowledge regarding the kinds of touch a specific patient might require, or prefer. Touch thus varies according to patient’s preference, but the degree to which this adaptation is possible, and successful, is dependent on the physiotherapist’s experience - Experience also gives PTs confidence regarding how to touch - Touch is intuitive and based on reciprocal feedback - While touch is used to assist, guide, and perform specific tasks, PTs simultaneously demonstrate caring attitudes and provide security through touch - Some touches thus have multiple intents (e.g. assisting + providing information; assisting + caring; assisting + security)	- Multiple intents of touch - Touch and experience - Touch as response - Touch as preference	II. Qualitative intents and meanings of touch III. Touch and its actors IV. Touch as waltz	“I’m comfortable in my skills as a therapist and my ability to get these people better. So, in that sense, I think that my caring touch has increased, while my solely assistive touch has decreased. I’ve gotten more comfortable with certain situations.” (177) “I think I use it, obviously for safety first of all for transfers and while walking. And then I think a lot of times I use it to make the patients feel a little more comfortable. A lot of times they are scared and may not understand what we are trying to do and just to help them feel a little more secure with what they are doing here” (178) “She would not move without me physically holding on to her. I knew she didn’t need me. I just wanted to get her started, get her going, show her that, with a sense of security, she can do this” (181)
Routasalo (1996) [75, 86]	- Non-necessary touching occurred in 99 nursing situations. 80 involved female patients, while only 19 involved male patients - 42% of nursing situations involving male patients had non-necessary touched; 58% for female patients - Non-necessary touches consisted of: short and long touch with the flat of the hand (*n* = 114), patting (*n* = 28), stroking (*n* = 16), holding hands, shaking, ticking with a finger, hugging (each, *n* < 5) - Non-necessary touching was often used in connection with verbal statements - Nurses often touched patients to encourage them towards doing something (stimulating independence) - Most touches (*n* = 107, 60%) were in the social zone, with 80 of them being on the shoulder and upper part of the back - 35 touches were in the consent zone, and 36 in the vulnerable zone	- Instrumental vs affective touch - Touch and gender - Touch on the body - Modalities of touch - Touch as persuasion	I. Quantitatively mapping touch II. Qualitative intents and meanings of touch III. Touch and its actors	N/A (quantitative)
Routasalo and Isola (1996) [75, 86]	- Non-necessary touch meant a great deal to the patient, since it establishes a closer relationship, and fosters mutual respect - Nurses’ touch was seen as warm, gentle and comforting - Nurses described touching as easy, natural and important, even moreso when they had worked with a patient for a long time - Touching is used to communicate emotional proximity and a listening attitude - Non-necessary touching requires reciprocity - Non-necessary touching should be intuitive	- Touch as humanising presence - Touch as communication and care	II. Qualitative intents and meanings of touch III. Touch and its actors	No quotes provided
Routasalo and Isola (1998) [183]	- Touch cannot be standardised, because each touch stems out of a unique grounded interaction - Nurses touch patients in order to help them, physically and emotionally - Nurses should avoid touching patients more than necessary, or if it is against their will - Some nurses show emotional and physical restraint by touching patients less - Nurses should verbalise touch intent before performing it - Nurses can touch patients to provide safety	- Touch as communication and care - Touch as embodied interaction	II. Qualitative intents and meanings of touch III. Touch and its actors	N/A (ethnographic)
Salzmann-Erikson and Eriksson (2005) [97]	- Patients long to be touched, as it gives them strength - Touching provides a sense of belonging and kinship - Touching is a natural element in human interaction - Feelings are intertwined with touching, therefore touch was seen as mediating emotions	- Touch as humanising presence - Touch as communication and care	II. Qualitative intents and meanings of touch	“Well, one kind of needs, when one has been ill and weak, then one needs someone who holds you and, you know, care […] but I still think that there is too little touching and commitment.” (847) “[through touch] then one feels a kind of friendship […] you feel a friendship and a sense [of] being connected to society” (848)
Tarantino et al. (2018) [95]	- Touch is used to perceive the other as a human being, establishing a relationship - Patients mention procedural touch is often too mechanic and de-personalised - Non-procedural touch usually provides a comforting feeling - Comforting touch is adapted to the needs of a patient, as well as being influenced by the nurse’s preferences and touching style - When a nurse does not establish a relationship based on consent and mutual proximity, even comforting touch can feel distressing - Touch can be used to establish mutual trust	- Touch as humanising presence - Touch as preference - Touch style - Touch as communication and care	II. Qualitative intents and meanings of touch III. Touch and its actors	“They only touch me for IV therapy. They plug me in, unplug me. They try not to hurt me but that’s about it.” (12 – 13) “We should strive to touch them to make them feel less lonely, abandoned […] protected. You make them feel secure, by showing your closeness and encouragement via touch.” (13) “With touch, you can break that distance between two people who don’t know each other” (14) “Being touched by someone who knows how to do their job feels good […] they know what to check and how to, and how adapt to my needs” (15)

## Appendix 5

**Table:** Confidence assessment (CERQual) and heterogeneity analysis

Finding	Summary	Studies included	CERQual confidence assessment	Notes	Heterogeneity
Touch and age	Age of both toucher and touched affects the amount of touch provided. The younger the personnel, the more they will touch; the older the patient, the more they will be touched	- Adomat and Killingworth (1994) [25] - Barnett (1972) [23, 77] - Cocksedge et al. (2013) [85] - Mulaik et al. (1991) [98]	Moderate confidence Methodological limitations: moderate Relevance: major concerns Coherence: no concerns Adequacy: moderate concerns	Only one study (Cocksedge et al. 2013) considers the question in relation to the context of the review questions (communication and affect), as well as being the only qualitative study. The finding is still reported given the homogeneity of the data, and the so-far under-investigated connections between age, gender and length of service	Adomat and Killingworth (1994) are the only one that found no relation between age and amount of touch, while also reporting that nurses with less than 2 years of experience touch more (see ‘Touch and experience’ finding). Such heterogeneity in the data could be explained by hypothesising that length of service could be seen as a more impactful variable over age—i.e. inexperienced practitioners could be seen as ‘younger’ irrespective of biological age. Such an explanation is reported on page 19
Touch on the body	Describes where on the body of the patient touch is concentrated. Hands, arms, head, trunk and legs generally touched the most, and considered ‘safe zones’	- Barnett et al. (1972) [23, 77] - Edwards (1998) [76] - Gale and Hegarty (2000) [78] - Hollinger and Buschmann (1993) - McCann and McKenna (1993) [20] - Routasalo (1996) [75, 86]	Moderate confidence Methodological limitations: moderate Relevance: moderate concerns Coherence: minor concerns Adequacy: minor concerns	Excellent variety of methods deployed; rich and coherent data. However, one study (Hollinger and Buschmann, 1993) reports data of poor quality (i.e. not fine-grained enough), while another (McCann and McKenna, 1993) seeks to understand patients’ experience, but only deploys non-participant observation	Barnett et al. (1972) report the face as being touched often, in contrast to all other studies. While this finding is reported, studies of higher methodological quality (i.e. more fine-grained and including patients’ and practitioners’ viewpoints) are given priority
Touch and gender	Strong interaction between the gender of the toucher and the amount of touch provided and its perception. Female staff are described as touching more, and patients prefer being touched by women	- Barnett et al. (1972) [23, 77] - Cocksedge et al. (2013) [85] - Gleeson and Higgins (2009) [99] - Jung and Fouts (2011) [66] - Kelly et al. (2020) [96] - McCann and McKenna (1993) [20] - Morris et al. (2014) [79] - Mulaik et al. (1991) [98] - O’Lynn and Krautscheid (2011) [100] - Routasalo (1996) [75, 86]	High confidence Methodological limitations: minor Relevance: no concerns Coherence: minor concerns Adequacy: minor concerns	Findings were mostly coherent, and varied methodologies were used to gather data concerning both the gender of the touched and the toucher	While Routasalo (1996) stresses that women are touched more than men, Mulaik et al. (1991) report that men are touched more often than women. Such heterogeneity might be partly explained by context—Routasalo’s research is carried out in Finland, where cultural understandings of touch might be different, and it might be less daunting to touch a woman. This hypothesis is reported on page 19 Jung and Fouts (2011) analysis showed no interaction between gender and the type of touch provided/received. However, such divergence might be explained by the stark difference in geographical and care context behind this reason (African informal care work)
Touch and experience	Touch might be influenced both by professional occupation, and length of service. Nurses tend to touch more than other medical professionals. Touch tends to be more communicative with experience	- Adomat and Killingworth (1994) [25] - Barnett (1972) [23, 77] - De Carvalho de Rezende (2015) [67] - Estabrooks and Morse (1992) [90] - Roger et al. (2002) [69]	Moderate confidence Methodological limitations: moderate Relevance: minor concerns Coherence: moderate concerns Adequacy: minor concerns	All but two studies (i.e. Estabrooks and Morse, 1992; Roger et al. 2002) reporting these findings are quantitative and are solely concerned with describing how the amount of touch changes with experience. They thus leave aside questions around how the communicative valence might change, something the two qualitative studies attempt to do by linking experience to the acquisition of style (see ‘Touch style’ finding)	Adomat and Killingworth (1994) found no relation between the length of service and the use of communicative touch, while the participants in Roger et al. (2002) study described how, with more experience, their communication via touch improves—which might imply less touching, but more affectively charged. Given the overall better quality of the paper, as well as the richness of the qualitative data provided, the argument put forward by Roger and colleagues seems more convincing, as we report on page 19
Touch and caring context	Touch might be deployed more in specific spatial contexts over others. Paediatrics, ITUs and care homes were found as spaces in which touch happens more often	- Barnett (1972) [23, 77] - Caris-Verhallen et al. (1999) [80]	Low confidence Methodological limitations: moderate Relevance: major concerns Coherence: major concerns Adequacy: moderate concerns	Both studies reporting this finding are quantitative and do not attempt to understand the communicative aims of touch. They also start from two very different contexts (intra-hospital comparison vs hospice care and domestic care), making a direct comparison and synthesis of the findings difficult. For these reasons, the findings have been excluded from the narrative synthesis, but the question of context has been reprised in the discussion	The findings of the two studies are not easily comparable, since one argues that touch is most used in ITU and paediatrics wards, while the other argues that touch is more used in care homes (compared to in-house interventions). This heterogeneity can be explained by the fact that the two studies are not comparing the same variables—one looked at intra-hospital differences, and other at inter-context differences
Touch as embodied interaction	Touch is configured as a dialogue mediated by the body. Such a dialogue allows transcending medical boundaries in which the patient is not merely an object of medical scrutiny but is rather an active subject, a full person implicated in a relational and embodied dynamic. Touch can thus function as a resource for sociality by regulating engagement and endowing physical engagements with meaning and agential power	- Bjorkbækmo and Mengshoel (2016) [70] - Consedine et al. (2016) [4] - De Luca et al. (2021) [91] - Eber (2018) [103] - Kelly et al. (2019) [89] - Kelly et al. (2020) [96] - Leonard and Kalman (2015) [104] - Mononen (2019) [68] - Routasalo and Isola (1998) [75, 86, 183]	High confidence Methodological limitations: moderate Relevance: no concerns Coherence: no concerns Adequacy: moderate concerns	All of the data reported by the studies in this finding directly respond to the central questions behind this review, particularly enriching the understanding of the phenomenon via extensive conceptual depth (introduction of concepts such as embodiment, haptic co-presence and tactile care). However, a few of the studies (e.g. Leonard and Kalman, 2015; De Luca et al. 2021) lack direct observation, or attempt to describe patients’ perspectives without actually discussing with patients (i.e. Consedine et al. 2016). In general, some of the findings might seem anecdotal given the ethnographic and phenomenological analytical lens, but the abundance of sources mitigate this risk. Richness and adequacy is however hampered by lack of depth in terms of empirically describing how such tactile interaction is structured – this point is reprised in the discussion	None are to be reported
Touch as response	Practitioners quickly respond and adapt to patients’ bodily reactions, changing the kinematic properties of their touch, e.g. tempo, and stroking force. This allows touch to become a dialogue at the moment, one in which the patient is a co-participant in a non-verbal engagement functioning via reciprocal feedback Such adaptations are usually made by gathering non-verbal information through one’s body (see ‘Touch as embodied interaction’ finding). Facial expressions, body language (closed/open) and eye contact (or lack thereof) were reported as cues used for effective responsive touch	- Bjorkbækmo and Mengshoel (2016) [70] - Bunzel et al. (2020) [72] - Consedine et al. (2016) [4] - De Luca et al. (2022) [83] - Estabrooks and Morse (1992) [90] - Gale and Hegarty (2000) [78] - Gleeson and Higgins (2009) [99] - Karlsson et al. (2022) [84] - Leonard and Kalman (2015) [104] - Roger et al. (2002) [69]	High confidence Methodological limitations: minor Relevance: no concerns Coherence: no concerns Adequacy: moderate concerns	All studies are methodologically sound in regards to attempting to understand practitioners’ point-of-view, but all of them stop at stressing that touch is adaptive, without going into details about how such adaptations take place (beyond some passing mentions, as elaborated in the finding ‘Touch and tempo’). This point is reprised in the discussion but contributes to a lower ‘adequacy’ score	None to be reported
Touch as risk	Touch can be misinterpreted by the receiver, particularly when specific gender dynamics are at play Touch was most often misinterpreted as controlling, disrespectful, creepy	- Bundgaard et al. (2011) [71] - Gleeson and Higgins (2009) [99] - Kelly et al. (2020) [96] - Mulaik et al. (1991) [98] - O’Lynn and Krautscheid (2011) [100]	High confidence Methodological limitations: none Relevance: no concerns Coherence: no concerns Adequacy: minor concerns	All studies directly engage with the central questions of this review, with some enriching the findings through theoretical exegesis (i.e. Kelly et al. 2020), and some bringing empirical richness and depth (i.e. Bundgaard et al. 2011; O’Lynn and Krautscheid, 2011). The number of studies is however limited	None to be reported
Multiple intents of touch	Instrumental touch was described as caring, affective and communicative, as a resource that simultaneously provides knowledge to practitioners while comforting patients	- Bundgaard et al. (2011) [71] - Cocksedge et al. (2013) [85] - De Luca et al. (2022) [83] - Karlsson et al. (2022) [84] - Leonard and Kalman (2015) [104] - Mononen (2019) [68] - Roger et al. (2002) [69]	High confidence Methodological limitations: none Relevance: no concerns Coherence: no concerns Adequacy: moderate concerns	All studies contributing to this finding are methodologically sound and engage with questions pertinent to the present review. Particularly, some of these studies use the data gathered to outline a preliminary critique of the division between affective and instrumental touch, recognising that affect cuts across said division. However, very few thick descriptions of how instrumental touch gains additional meanings are provided, with most studies stopping at enlisting the different communicative aims co-existing in a medical touch procedure (e.g. Roger et al. 2002)	None are to be reported
Touch as humanising presence	Touch can create a sense of presence, enhancing the feeling of being cared for by establishing a socio-affective dimension to the medical encounter. Touch can thus function to acknowledge patients’ vulnerability, while also reaffirming the practitioner’s physical and emotional proximity through a compassionate and understanding stance which fosters mutual trust	- Bundgaard et al. (2011) [71] - De Luca et al. (2021) [91] - Kelly et al. (2020) [96] - Mononen (2019) [68] - Routasalo and Isola (1996) [75, 86] - Salzmann-Erikson and Eriksson (2005) [97] - Tarantino et al. (2018) [95]	High confidence Methodological limitations: minor Relevance: no concerns Coherence: no concerns Adequacy: no concerns	While one study (Salzmann-Erikson and Eriksson, 2005) presents little data to ground its findings, the rest of the studies present rich and varied findings gathered through sound methods The finding gains in coherence and relevance when connected to data presented in other findings (e.g. ‘Touch as preference’) which show how touch can also be de-humanising and isolating when not deployed properly (i.e. without ‘care’)	None to be reported
Touch as preference	The amount of touch provided is dictated by the individual preferences of both the practitioner and the patient. Practitioners thus need to be able to understand patients’ preferences and tailor their touch to these, since patients might feel powerless when their touch preferences are not respected On the other hand, practitioners might have preferences too, thus getting embarrassed when touching areas they might not be comfortable touching, or reducing touch when they feel insecure Cultural background might influence touch preferences	- Bundgaard et al. (2011) [71] - De Luca et al. (2021) [91] - De Luca et al. (2022) [83] - Dobson et al. (2004) [87] - Edwards (1998) [76] - Estabrooks and Morse (1992) [90] - Gleeson and Higgins (2009) [99] - Kelly et al. (2019) [89] - O’Lynn and Krautscheid (2011) [100] - Pedrazza et al. (2018) [182] - Roger et al. (2002) [69] - Tarantino et al. (2018) [95]	Moderate confidence Methodological limitations: moderate Relevance: minor concerns Coherence: minor concerns Adequacy: moderate concerns	Similarly to the ‘Touch as response’ finding, most studies reporting this finding seem more concerned with proving that preferences matter, without attempting to explore and schematise different types of preferences and the role that different variables might play. For instance, cultural background and upbringing is often brought up as a factor influencing one’s preferences towards touch; however, beyond anecdotal evidence from individual practitioners, no effort is made to empirically outline different cultural attitudes towards touch. This might also be partly caused by the fact that, methodologically, none of the studies reporting this finding took a cross-cultural analysis approach. Such an omission regarding the exploration of cultural background as a variable impacting the communicative function of touch has been reported in the discussion	None are to be reported
Touch as communication and care	Touch is described as a tool to (re)affirm empathy, since it can make patients feel cared for, nurtured and supported; it thus provides a humanising dimension to healthcare Touch can thus be seen as a tool for non-verbal communication, particularly effective in comforting and reassuring patients, while also breaking boundaries between practitioner and client by opening up an affective space in which both parties can feel safe and calm Touch in this sense is described as a mediator for emotions since feelings are intertwined with bodily expressions of it: touch can thus mitigate negative emotions on the other side, functioning as a form of emotional containment, for instance when a doctor breaks down a bad news to a patient, and does so by holding their hand	- Bundgaard et al. (2011) [71] - Caris-Verhallen et al. (1999) [80] - Cocksedge et al. (2013) [85] - Consedine et al. (2016) [4] - De Carvalho de Rezende et al. (2015) [67] - De Luca et al. (2021) [91] - Dobson et al. (2004) [87] - Gleeson and Higgins (2009) [99] - Karlsson et al. (2022) [84] - Kelly et al. (2019) [89] - Mulaik et al. (1991) [98] - Pratt and Mason (1984) [88] - Routasalo and Isola (1996) [75, 86] - Routasalo and Isola (1998) - Salzmann-Erikson and Eriksson (2005) [97] - Tarantino et al. (2018) [95]	High confidence Methodological limitations: minor Relevance: minor concerns Coherence: no concerns Adequacy: minor concerns	All papers present an adequate methodology to answer the themes within this finding, with most providing ample quotes and/or thick descriptions, albeit sometimes veering on the anecdotal. For example, Cocksedge et al. 2013 start with the aim of analysing medical touches, but end up focusing more on touches that are decidedly not medical, and are just happening within a medical context	None are to be reported
Touch, vision and collaboration	Nurses must be able to always see a patient’s face, and pay attention to their facial expressions as they are mobilising them. In certain scenarios (e.g. partial visual GM), a nurse might require a second nurse present acting as their ‘eyes’, checking on the patient’s reactions and relaying them to the nurse touching them	- Bunzel et al. (2020) [72]	Low confidence Methodological limitations: minor Relevance: minor concerns Coherence: N/A Adequacy: major concerns	While an interesting finding was reported in a high-quality study, the theme emerged only from one ethnographic vignette from one study with an already very small pool of participants. From a relevance perspective, the question of collaboration risked also unnecessarily complicating the narrative drafted from all other data gathered (i.e. touch as a dyadic dialogue), since it complexifies touch as possibly multi-agential. For these reasons, this finding has been only briefly mentioned in relation to patients’ preferences	N/A
Affective vs instrumental touch	Instrumental touch was described as overwhelmingly more deployed than affective touch	- Caris-Verhallen et al. (1999) [80] - McCann and McKenna (1993) [20] - Morris et al. (2014) [79] - Mulaik et al. (1991) [98] - Routasalo (1996) [75, 86]	Moderate confidence Methodological limitations: moderate Relevance: moderate concerns Coherence: minor concerns Adequacy: moderate concerns	All the studies describing this finding are quantitative in nature. In this sense, they do not acknowledge how instrumental touches could have a communicative and affective component, and vice versa, as picked up by most other studies either implicitly, or explicitly. Despite its evident limitations, this finding has been included in the narrative synthesis to provide an initial template on how to rigorously analyse touch (see discussion), as well as to present how studies on medical touch greatly evolved over time (most of these studies were carried out in the 1990s), and how they vary across disciplines (quantitative vs qualitative)	While all studies agree on the fact instrumental touch is more often deployed, the amount of affective touch usage (in percentage and absolute terms) widely varied across studies—i.e. going from as low as 4% of recorded touches (McCann and McKenna, 1993), from as high as 40% of touching encounters (Caris-Verhallen et al. 1999) Such heterogeneity in reporting could be attributed to differences in counting methodologies, or in actual differences in touch expression in different professional or cultural contexts. More crucially, however, these discrepant results might also be seen as an indicator of how feeble the definition of expressive touch is, as further discussed in other findings Such explanations are presented in the narrative synthesis on page 16
Touch style	The amount and type of touch provided is highly dependent on the practitioners’ style. The style here is understood as different from a mere preference; rather, it is an embodied and highly personal way of being and understanding touch, one that is acquired in time and through different professional and non-professional experiences	- Caris-Verhallen et al. (1999) [80] - Estabrooks and Morse (1992) [90] - Dobson et al. (2004) [87] - Kelly et al. (2019) [89] - Morris et al. (2014) [79] - Tarantino et al. (2018) [95]	Moderate confidence Methodological limitations: minor Relevance: no concerns Coherence: minor concerns Adequacy: moderate concerns	Some of the studies (i.e. Caris-Verhallen et al. 1999; Morris et al. 2014) are quantitative, and the idea of style influencing touch is merely hypothesised—although this is not clearly stated by the authors. The qualitative studies describing this finding often lacked enough empirical material to substantiate the claims, or to connect such claims to robust theoretical thinking. Contrary to this negative adequacy evaluation, Estabrooks and Morse (1992) instead provide an extensive analysis of touch style development in healthcare professionals	Morris et al. (2014) argue that style differences play a role in expressive/affective touches, while instrumental touches remain the same irrespective of personal style. All other studies seem to implicitly or explicitly (e.g. Tarantino et al. 2018) refute this Given such a claim was made by only one study out of six, and, more importantly, it was based on observational data, we attribute such heterogeneity to poor interpretation of the data
Touch and time	Affective touch is said to emerge with time when a relationship between patient and practitioner is established	- Gale and Hegarty (2000) [78]	Very low confidence Methodological limitations: major Relevance: moderate concerns Coherence: N/A Adequacy: major concerns	Only one study reported this finding, and without further qualifying what ‘building rapport through time’ might look like. This is partially imputable to the fact the study only deployed non-participant observation, so participants’ perspectives are not emerging. For these reasons, the finding has been excluded from the narrative synthesis	N/A
Touch and tempo	Accommodating the tempo of the touch to patients’ preferences is described as crucial to providing a dialogical and affective valence to the touch interaction, but its effective deployment can prove challenging. In fact, a touch that is too fast can be perceived as the practitioner being embarrassed, while a touch that is too slow can be felt as creepy	- Bjorkbækmo and Mengshoel (2016) [70] - Leonard and Kalman (2015) [104] - O’Lynn and Krautscheid (2011) [100]	Moderate confidence Methodological limitations: moderate Relevance: no concerns Coherence: no concerns Adequacy: moderate concerns	While an interesting finding was extracted through appropriate methods and theoretical analysis, descriptive analysis in the style of ethnographic vignettes could have benefitted the presentation of such an embodied, experiential finding, allowing us to better understand both the affective and kinematic dimensions of this adaptation. This is particularly relevant to studies that solely relied on interviews (e.g. Leonard and Kalman, 2015). Such a methodological and empirical omission is reported in the discussion	None are to be reported
Touch as persuasion	Touch can be used as means of persuasion and encouragement, used to physically reaffirm verbalised orders and maintain attention to a given social scenario	- Edwards (1998) [76] - Mononen (2019) [68] - Routasalo (1996) [75, 86]	Moderate confidence Methodological limitations: major Relevance: minor concerns Coherence: no concerns Adequacy: moderate concerns	While the findings seem to be homogeneous across multiple studies, none of the studies reporting them deployed interviews or any other methods to gather patients’ or practitioners’' first-hand experience. In this sense, nothing is said regarding how persuasion is understood and felt, as well as hampering the overall descriptive and analytical depth of the data reported	None are to be reported
Modalities of touch	This finding describes the modalities used to engage affectively with patients via touch. Stroking, embracing, patting and short and long touches were the most reported modalities	- Mononen (2019) [68] - Routasalo (1996) [75, 86] - Gale and Hegarty (2000) [78]	Low confidence Methodological limitations: moderate Relevance: moderate concerns Coherence: major concerns Adequacy: major concerns	Only three papers attempted to describe in detail the qualities of the touch itself. They did so in very contrasting terms. Moreover, the two papers that described more attentively and quantitatively modes of touch did not attempt to understand their communicative meaning, while the other focused on affect and communication, but only anecdotally described touch gestures. The findings are still presented in the narrative synthesis, but the discussion strongly advocates for more attention to said aspects	Mononen (2019) argues that stroking and embracing are more common, while Routasalo (1996) pinpoints patting and short touches with the flat of the hand as the most common touches, and Gale and Hegarty (2000) point to stroking and rubbing as the most used techniques. Such heterogeneity might be because of different caring contexts. However, given only three papers report this finding, and they fundamentally disagree, the overall confidence in this finding is low. This explanation is reported at page 16
Light touch and indifferent touch	Light touch is described as being more used by nursing staff, while indifferent touch is mostly used in technical procedures	- De Carvalho de Rezende et al. (2015) [67]	Very low confidence Methodological limitations: major Relevance: moderate concerns Coherence: N/A Adequacy: major concerns	Only one study of poor theoretical and methodological quality brought up this division between light and indifferent touch, without anchoring the analysis in the literature extensively. The finding has thus been excluded from narrative synthesis	N/A
Touch and sexual orientation	Male patients described being touched by a gay male nurse as potentially embarrassing	- O’Lynn and Krautscheid (2011) [100]	Very low confidence Methodological limitations: major Relevance: moderate concerns Coherence: N/A Adequacy: major concerns	This finding has been mentioned only in one study, without providing any quotes. The finding has thus been excluded from narrative synthesis	N/A
Directionality of touch	Patients are expected to be touched by nurses, but not the reverse	- Edwards (1998) [76]	Low confidence Methodological limitations: minor Relevance: moderate concerns Coherence: N/A Adequacy: minor concerns	While the finding is reported by only one study, the data provided are rich enough to warrant inclusion in the narrative synthesis, while making explicit reference to this limitation (page 16). Moreover, the relevance to the overall aim of the study is hampered by a limited interest in investigating the counter-issue to these expectations regarding the directionality of touch—how do practitioners feel when unexpectedly touched by patients?	N/A

## Appendix 6

**Table:** Appraisal and risk of bias analysis (MMAT)

Study	Methodology	MMAT Score (out of 5)	Notes
Adomat and Killingworth (1994) [25]	Mixed methods	4	Qualitative part of study of lesser quality (e.g. no quotes provided)
Barnett (1972) [23, 77]	Quantitative (descriptive)	4	No interaction analysis performed
Bjorkbækmo and Mengshoel (2016) [70]	Qualitative	4	Observation and interviews are used disjointly
Bundgaard et al. (2011) [71]	Qualitative	5	//
Bunzel et al. (2020) [72]	Qualitative	4	Interpretations are often grounded in one or two ethnographic episodes only
Caris-Verhallen et al. (1999) [80]	Quantitative (descriptive)	5	//
Cocksedge et al. (2013) [85]	Qualitative	5	//
Consedine et al. (2016) [4]	Qualitative	3	Configures touch as a dialogue, but only look at practitioners’ perspective
De Carvalho de Rezende et al. (2015) [67]	Quantitative (descriptive)	3	Unclear methods. Small and possibly not representative sampling pool
De Luca et al. (2021) [91]	Qualitative	5	//
De Luca et al. (2022) [83]	Qualitative	4	Interesting theoretical points, but not sufficiently substantiated by data
Dobson et al. (2004) [87]	Qualitative	3	Data are of poor quality. Lack of coherence between data and theory
Eber (2018) [103]	Qualitative	4	Interpretations often grounded in one or two ethnographic episodes only
Edwards (1998) [76]	Qualitative	5	//
Estabrooks and Morse (1992) [90]	Qualitative	4	Extent to which theoretical analysis is based on data not clear (no quotes)
Gale and Hegarty (2000) [78]	Quantitative (descriptive)	3	Sampling strategy not clear
Gleeson and Higgins (2009) [99]	Qualitative	4	Contrast between some theoretical assumptions and data provided
Hollinger and Buschmann (1993) [181]	Quantitative (descriptive)	4	Measurements are not fine-grained enough
Jung and Fouts (2011) [66]	Quantitative (descriptive)	5	//
Karlsson et al. (2022) [84]	Qualitative	5	//
Kelly et al. (2019) [89]	Qualitative	4	While attempting to understand all touch, it focuses on non-medical touch
Kelly et al. (2020) [96]	Qualitative	5	//
Leonard and Kalman (2015) [104]	Qualitative	5	//
McCann and McKenna (1993) [20]	Mixed methods	3	Poor integration between quantitative and qualitative methods
Mononen (2019) [68]	Qualitative	4	Research aim could have benefitted from use of interviews
Morris et al. (2014) [79]	Quantitative (descriptive)	5	//
Mulaik et al. (1991) [98]	Quantitative (descriptive)	4	No interaction analysis performed
O’Lynn and Krautscheid (2011) [100]	Qualitative	5	//
Pedrazza et al. (2018) [182]	Quantitative (descriptive)	5	//
Pratt and Mason (1984) [88]	Quantitative (descriptive)	5	//
Roger et al. (2002) [69]	Qualitative	5	//
Routasalo (1996) [75, 86]	Quantitative (descriptive)	4	Uneven sample pool (i.e. mostly female patients)
Routasalo and Isola (1996) [75, 86]	Qualitative	3	Quotes not provided. Unclear connections findings/interpretation
Routasalo and Isola (1998)	Qualitative	5	//
Salzmann-Erikson and Eriksson (2005) [97]	Qualitative	3	Sparse data points. Could have benefitted from observation data
Tarantino et al. (2018) [95]	Qualitative	5	//
